# Structural Motifs at the Telomeres and Their Role in Regulatory Pathways

**DOI:** 10.1021/acs.biochem.4c00023

**Published:** 2024-03-14

**Authors:** Abeer F R Alanazi, Gary N Parkinson, Shozeb Haider

**Affiliations:** † UCL School of Pharmacy, University College London, London WC1N 1AX, United Kingdom; ‡ UCL Centre for Advanced Research Computing, University College London, London WC1H 9RN, United Kingdom

## Abstract

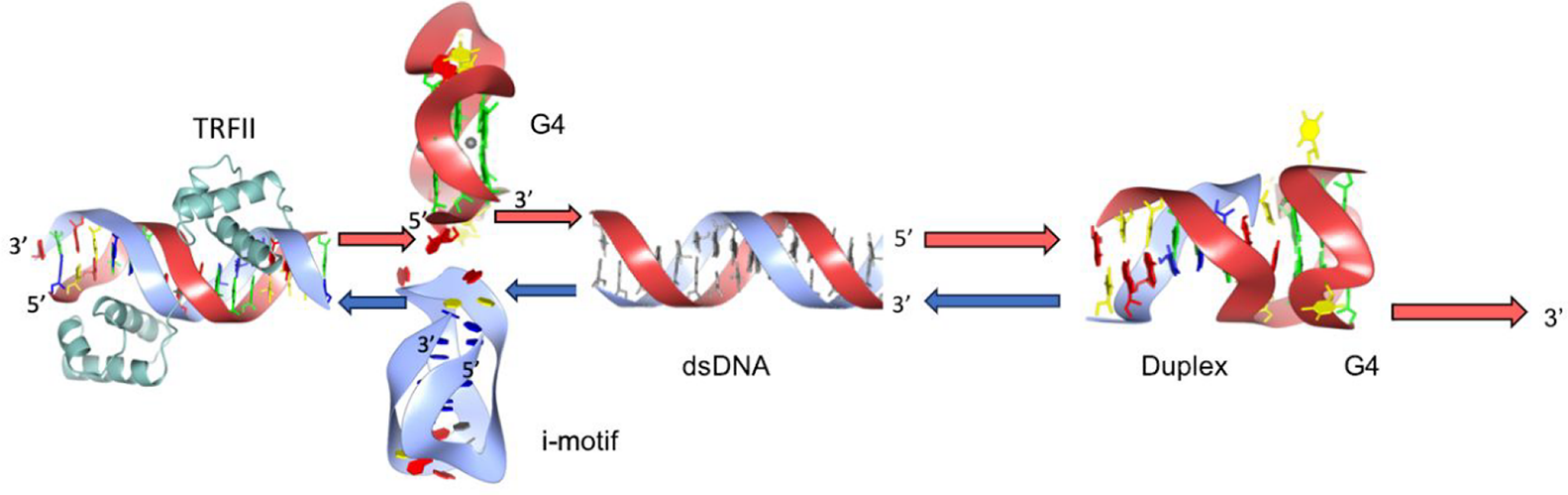

Telomeres are specialized structures, found at the ends of linear chromosomes in eukaryotic cells, that play a crucial role in maintaining the stability and integrity of genomes. They are composed of repetitive DNA sequences, ssDNA overhangs, and several associated proteins. The length of telomeres is linked to cellular aging in humans, and deficiencies in their maintenance are associated with various diseases. Key structural motifs at the telomeres serve to protect vulnerable chromosomal ends. Telomeric DNA also has the ability to form diverse complex DNA higher-order structures, including T-loops, D-loops, R-loops, G-loops, G-quadruplexes, and i-motifs, in the complementary C-rich strand. While many essential proteins at telomeres have been identified, the intricacies of their interactions and structural details are still not fully understood. This Perspective highlights recent advancements in comprehending the structures associated with human telomeres. It emphasizes the significance of telomeres, explores various telomeric structural motifs, and delves into the structural biology surrounding telomeres and telomerase. Furthermore, telomeric loops, their topologies, and the associated proteins that contribute to the safeguarding of telomeres are discussed.

Eukaryotic cells contain linear chromosomes that are encased by a nucleoprotein complex at each end called a telomere.^1^ The hexanucleotide repeats found within telomeric DNA are linked to various proteins that bind to both telomeric double-stranded DNA (dsDNA) and single-stranded DNA (ssDNA), through direct or indirect interactions. These proteins come together to form the protective protein telomere cap.^2^ Telomeres play a crucial role in stabilizing the ends of chromosomes, and their protective function may hinge on whether they are in an “uncapped” or “capped” state.^2,3^ For an extended period, it was believed that the telomeres were transcriptionally inactive. Nevertheless, recent findings have revealed that telomeric DNA is often transcribed into telomeric repeat-containing RNA (TERRA).^4^ Telomeric RNA is a newly emerging component in telomeric function that could be an important element of telomere machinery.^2^ Previously, studies focused on telomeric DNA and its linked proteins. However, the identification of TERRA RNA at the chromosome ends has the potential to provide fresh perspectives and enrich our existing understanding.^5^ The proteins present at the telomeres possess a unique structure that allows them to oversee and safeguard DNA, making them integral to numerous biological processes. Additionally, telomeres play a role in regulating gene expression and function as a molecular timer, governing the replicative capacity of human cells.^6^ In proliferating cells that lack functional telomerase, telomeres shorten with each mitotic division, and the cells finally die. The telomeric DNA and TERRA RNA structural motifs are discussed in detail in this Perspective along with their roles in regulatory pathways.

## Structure and Function of Telomeres

Telomeric DNA is composed of repetitive sequences located at the termini of chromosomes. This characteristic is observed in a diverse range of eukaryotic species. The guanine-rich (G-rich) sequences consist of tandem repeats of (TTTTGGGG)n in lower eukaryotes such as Oxytricha, or (TTGGGG)n in Tetrahymena and (TTAGGG)n in vertebrates.^7,8^ Telomeric sequences in human somatic cells usually range in length from 10 to 15 kb.^9^ Contrary to the rest of the double-stranded telomeric DNA, the G-rich strand has a 5′ to 3′ strand orientation, and the 3′ end strand extends past the complementary C-rich stand in a single-strand overhang.^10^ This overhang length varies depending on the species and is typically 50–200 nucleotides long in humans.^11^ Recent studies have confirmed the presence of several proteins that are essential in maintaining the integrity of the telomeric DNA (Table 1). Telomeres can therefore be defined as ribonucleoprotein complexes present at the ends of the chromosomes.

**Table 1:** A Summary of the Telomere-Associated Proteins Discussed in This Review

Telomere-associated proteins	Role at telomeres	PDB id (if available)	Reference
Telomerase	Extension of telomeric DNA	7QXB	(17, 24, 27)
The shelterin complex	Protects and regulates telomeres. Consists of six proteins TPP1-POT1-TRF1-TRF2-TIN2-RAP1		(34, 35)
TPP1 (POT1 interacting protein)	Interacts with PO1 and TIN2. Recruits telomerase to telomeres and is in direct contact with telomerase	5UN7,5H65,7TRE,5XYF	(26, 36−38)
POT1 (protection of telomeres)	Recognizes the 3′ single strand and binds to ss-ds DNA junction, prevents telomere instability	8SH1,7S1O,7S1T,7S1U	(27, 30, 32, 33, 39)
TRF1 (telomere repeat binding factor)	Recognizes dsDNA TTAGGG sequences	8OX1,1W0T	(29, 40−43)
TRF2 (telomere repeat binding factor)	Binds to and promotes the development of T-loops	1W0U,5XYF	(29, 37, 41, 43, 44)
TIN2 (TRF interacting nuclear)	Interacts with TPP1, TRF proteins and acts as a bridging unit	5XYF	(37, 45, 46)
RAP1 (repressor/activator protein)	Dependent on TRF2 for telomere binding, inhibits DNA repair	3K6G	(47−50)
ssDNA binding CST complex	Consists of three proteins CTC1-STN1-TEN1	8SOK	(25, 51, 52)
CTC1	Controls access of telomerase, prevents G-overhang extension, involved in telomere length homeostasis	6W6W	(25, 53, 54)
STN1	Binds to ssDNA and protects telomeres from DNA degradation	4JOI	(25, 53, 55)
TEN1	Required for DNA polymerase α-mediated C-strand synthesis	4JOI	(25, 51, 52, 55, 56)
DNA2 helicase	Interacts with TRF1/2 in shelterin complex, removes telomeric G4	5EAX,5EAN	(57−59)
Pif1 helicase	Unwinds G4, inhibits telomerase activity at telomeres	6HPT,6L3G,7OAR	(60−65)
FANCJ (Fanconi anemia complementation group J) helicase	Involved in homologous recombination, DNA damage repair, G4 resolution, and maintaining genomic stability		(66, 67)
StyRecQL (stylonychia RecQ-like) helicase	Resolution of telomeric G4		(68)
TLS (translocated in liposarcoma)/FUS (fused in sarcoma) proteins	Binds to telomeric G4 DNA and TERRA		(69)
HMGB1 (high mobility group B1) protein	Binds to noncanonical DNA structures like G4, hemicatenated DNA loops, and four-way junctions	4QR9	(70−72)
Gen1 (genetic endonuclease)	Resolves HJ at T-loops	5T9J	(73, 74)
SLX1/4 (structure-specific DNA binding protein required for maintenance of genome stability X) endonuclease	Resolves HJ at T-loops	7CQ4	(75, 76)
RTEL1 helicase	Unwinds T-loops, promotes telomere replication	7WU8	(77−79)
RecQ helicase complex	Consists of SGS1-TOP3-RMI1-MPH1-SRS2 proteins. Resolves D-loop, unwinds G4	2WWY,6CRM	(80−83)
ATM (Ataxia Telangiectasia mutated) kinase	Involved in dsDNA breaks	8OXP	(84−86)
ATR (Ataxia Telangiectasia and RAD3) kinase	Involved in ssDNA damage	5YZ0	(84, 85, 87)
MRN complex	Consists of MRE11-RAD50-NBS1 proteins. Recognizes ds breaks, primes DNA ends for repair, activating ATM, implicated in nonhomologous end joining and homologous recombination	8BAH,3AV0,3QKU	(88−91)
RAD51	Facilitates strand exchange during HR, involved in D-loops and recruitment of TERRA via R-loops	5H1B	(80, 92−94)
RNase H1 and H2	Prevents R-loop accumulation	2QK9,3P56	(95−97)
ATP-dependent DNA helicase senataxin	Prevents R-loop accumulation		(98, 99)
DHX9 helicase	Unwinds R-loops and G4	8SZP	(100, 101)
EST1A (ever shorter telomeres)/SMG6	Regulates telomerase via TERRA, involved in nonsense-mediated decay process	2HWW	(102−105)
RAD51AP1 (RAD51-associated protein)	Involved in R-loop and D-loop formation, role in ALT pathways		(106)
BRCA1 (p220)	Deals with ssDNA damage at R-loop termination sites		(107, 108)

In the process of lagging strand DNA synthesis, the terminal regions of linear DNA are unable to achieve complete replication. This arises from the intrinsic asymmetry inherent in semiconservative DNA replication, a phenomenon commonly referred to as the “end-replication problem.”^12^ As normal somatic cells divide, telomeric repeats gradually shorten with every replicative cycle at a rate of 50–200 bp per cell division. Soon after, the cells enter a state of irreversible growth inhibition and eventually die via cellular apoptosis.^8^ The shortening of telomeric DNA can be viewed as a molecular clock that marks the beginning of cellular senescence.^9^ This biological phenomenon has been linked to cellular immortality and aging because immortalized cells’ telomeric DNA does not shrink following division.^13^

As a result, it is essential to preserve both the length of the telomeres and the presence of the single-stranded overhang for the stability of chromosomes and the viability of cells. Failure to do so results in end-to-end fusion events and erosion that lead to genomic instability and cell senescence.^14^ Furthermore, telomeres have also been linked to sister chromatid pairing during mitosis, homologous meiotic synapses, and the development of nuclear regions that may be critical for transcriptional regulation.^15^ When DNA breaks are detected, DNA damage surveillance proteins activate enzymes that break down DNA or fuse chromosomal ends. These biological processes highlight the importance of telomeres in the protection of chromosomal ends.

In addition to protecting the ends of DNA strands, telomeres carry out additional vital tasks such as controlling the expression of genes that are either close to the telomeres (called TPE) or far from them (called TPE over long distances, or TPE-OLD).^16^ The minimal length of telomeric DNA repeats and the efficiency of the related protein complexes are two factors that strictly control the function of telomeres.^17^ Furthermore, it is believed that appropriate telomere function is aided by higher-order DNA conformations like the G-quadruplexes (G-rich, four-stranded nonhelical structures) and T-loop.^12^ Moreover, telomeric chromatin plays a crucial role in signaling, maintaining telomeres, and controlling telomere function; nevertheless, many of the specific molecular mechanisms and structures of human telomeric chromatin remain unclear. Additionally, RNA polymerase II transcribes a lengthy noncoding RNA called telomeric repeat-containing RNA (TERRA) from telomeric DNA in telomeric regions.^18^ TERRA has been linked to the regulation of telomerase, the arrangement of heterochromatin at telomeres, the control of gene expression, and the DNA damage response (DDR) that is brought on by telomere malfunction.^19^

## Maintenance of Telomeres

Recombination and retrotransposition are two examples of the diverse processes that have evolved in various cells to stop the progressive degradation of telomeres.^20,21^ Telomerase, a specialized enzyme present in eukaryotes, is an RNA-dependent DNA polymerase complex that helps in the maintenance of telomere length by synthesizing telomeric DNA sequences.^8^ Besides maintaining telomeric length in the germline or rapidly dividing cells, telomerase also plays a key role in tumorigenesis and is a hallmark of cancer.^14^

Telomerase is a ribonucleoprotein multicomplex composed of a catalytic protein subunit (hTERT), also known as the TERT reverse transcriptase, and an RNA moiety (hTR). The expression of hTERT protein is not typically observed in normal somatic cells, whereas TER is not only present in telomerase negative cells but is effectively recruited into a fully functional ribonucleoprotein complex upon the introduction of hTERT *in vitro.*(14) hTR is a ubiquitously expressed RNA component that serves as a template for the insertion of TTAGGG repeats to the ends of chromosomes, thereby aiding in the catalysis, localization, and assembly of the telomerase.^22^ Telomerase almost universally provides the molecular basis for unlimited proliferative potential. Telomerase is present in ∼85% of all cancer cells and absent in normal somatic cells.^23^

**Figure 1:**
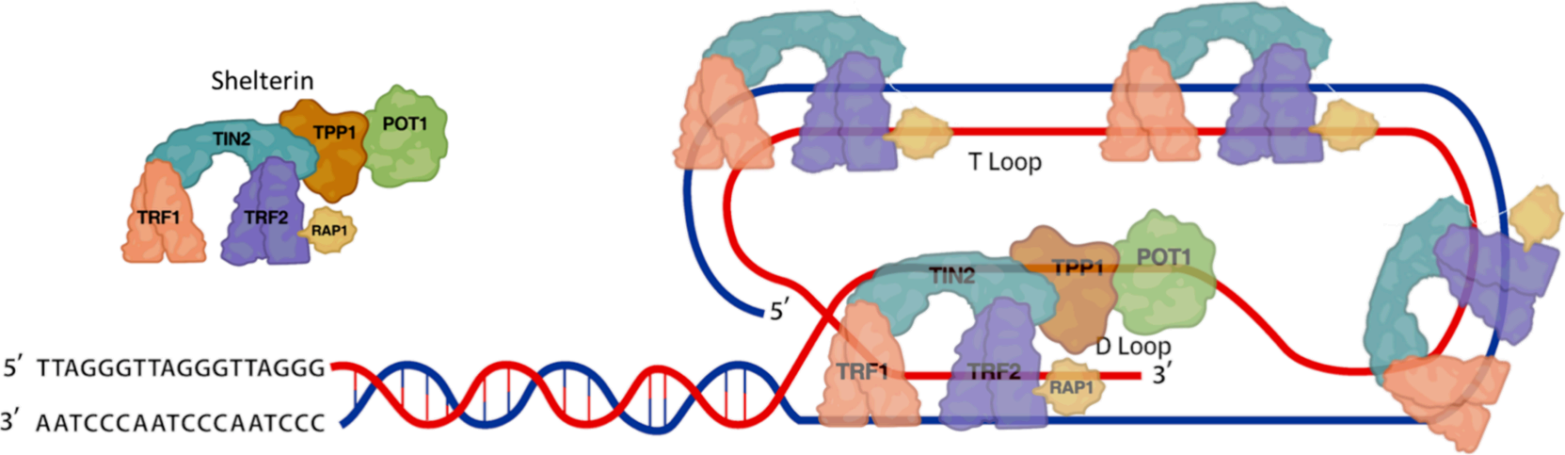
Human telomeres associate with the six-protein shelterin complex. A homodimeric protein called TRF1 interacts with the double-stranded DNA (top strand: TTAGGG). TRF2 aids in the T-loop formation. RAP1, a protein connected to TRF2, prevents DNA repair. POT1 interacts with ssDNA. When TPP1 is lost, POT1 function is hampered. A protein called TIN2 interacts with the TPP1-POT1 complex, TRF1, TRF2, and TIN2.

Telomerase differs from other reverse transcriptases in that it carries its template RNA for telomeric DNA synthesis. The RNA component consists of 451 nucleotides. This RNA contains a sequence that is complementary to about 11 bps in humans, acting as a template for telomere replenishment.^21^ The template region is longer than the telomeric repeat that it encodes. The longer template plays an important role in both alignment and elongation. By using base-pairing, a piece of the template aligns with the primer 3′ section, and elongation replicates it to the 5′ end. No matter where synthesis begins within the template area, the length of the template guarantees complete replication of the telomeric repeat sequence. The elucidation of telomerase, TER, and its associated protein complexes by the use of cryo-EM places the individual elements in a wider structural context.^24−27^ The structures are now available of the human telomerase with telomerase RNA (TER) bound to the shelterin protein TPP1 (PDB ID 7TRE)^26^ and a larger complex of telomerase-DNA-TPP1-POT1 (PDB ID 7QXB)^27^ that spatially places the key telomere binding proteins in context for recruitment of telomerase to the telomere and its processivity (Figure 1).

## Telomere Binding Proteins

Telomerase can bind ssDNA *in vitro* and extend primer sequences in the presence of NTPs; but *in vivo*, the appropriate substrate for telomerase activity is not naked DNA repeats but the shelterin complex, which is a collection of six proteins (Figure 1).^28^ Telomeric DNA sequences are directly recognized by the three proteins, TRF1, TRF2, and POT1. However, a shelterin complex is created when three more proteins, TIN2, TPP1, and RAP1, associate together. These core proteins act to recruit additional components to the telomere to create multiprotein complexes that help regulate telomere maintenance.^28^

**Figure 2:**
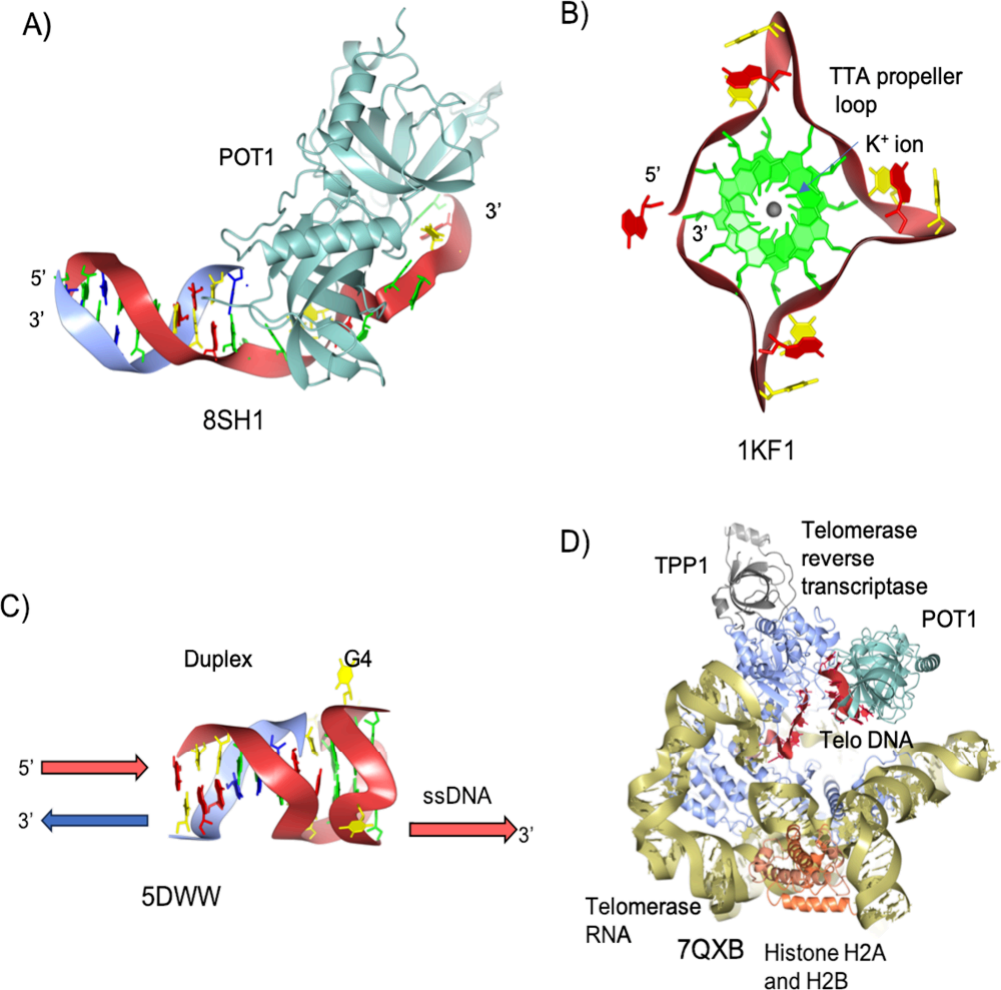
Structures found at the human telomeres. (A) Structure of POT1 (cyan) bound to duplex/single-stranded interface and the 3′ end of unfolded telomeric ssDNA; (B) the crystal structure of the propeller topology of human telomeric DNA G4; (C) structural model of the G4-duplex DNA interface; (D) structure of telomerase holoenzyme illustrating the reverse transcriptase (ice blue)-telomerase RNA (khaki)-TPP1 (gray)-POT1 (cyan)-histone H2A/B (orange)-telomeric DNA (red). Red arrows show the G-rich DNA direction, and blue arrows show the C-rich DNA direction. The corresponding PDB identifiers are listed below each subfigure.

Telomeric repeat-binding factors 1 and 2 (TRF1 and TRF2) directly attach to dsDNA TTAGGG sequences through a DNA binding domain located at the C-terminal region of the protein.^29^ This provides a shielding effect on the chromosomal ends, thereby preventing an incorrect DNA damage response. TRF1 has acidic amino acids close to its N-terminus, while TRF2 has a basic region rich in Gly/Arg. They bind DNA as homodimers or oligomers by homotypic interactions in the TRF homology (TRFH) domain. These proteins connect to arrays of the telomeric sequence TAGGGTTAG with remarkable sequence selectivity. Nonetheless, the protection of telomeres 1 (POT1) protein recognizes the 3′-single-stranded overhang at the end of the chromosomal dsDNA.^30^ POT1 protein is present in organisms such as the microsporidia, plants, mammals, and fission yeast.^31^ It is a highly conserved protein that is essential for the regulation and maintenance of telomerase. Additionally, POT1 structural domains have been identified either alone or in complexes, such as POT1a bound to dsDNA with a GTTAGG repeat 3′-overhang (PDB ID 8SH1).^32^ Another is the c-terminal domain (POT1c) (PDB 7S1O, 7S1T, and 7S1U).^33^ The complex describes how POT1a functions to safeguard the key interaction site known as the ds-ssDNA junction. This structural configuration emphasizes how essential it is to cap the phosphorylated 5′ C-rich strand of the junction, inhibit RPA (replication protein A) loading, and restrict ATR (Ataxia telandiectasia and RAD3) recruitment (Figure 2A). Furthermore, the interactions between the POT1 OB/TPP1 binding domains with the shelterin complex bind the ssDNA overhang to the telomeric dsDNA.

Moreover, TRF1- and TRF2-interacting nuclear protein 2 (TIN2) are used by TPP1-POT1 heterodimers to link the duplex section of the telomeres to the single-stranded overhang.^45^ TIN2 interacts with TRF1 at its C-terminus, and it binds to a hinge domain at TRF2’s N-terminus. Using a third protein interaction site located at its N-terminus, TIN2 binds to TPP1 and forms a complex. Thus, via binding to TRF1, TRF2, and TPP1, TIN2 plays a crucial part in the shelterin complex.^28,34^ Repressor/activator protein 1 (RAP1) in humans is dependent on TRF2 for telomere binding, since it is not capable of binding DNA.^48^ Through its C-terminal domain, RAP1 interacts with a tiny helical area in TRF2’s hinge domain to form a complex.^47^

TIN2 and TPP1 (POT1 interacting protein 1) interacts with POT1 and TIN2 via the POT1-binding domain and the C-terminal of TIN2.^46^ POT1’s association with telomeres is contingent upon its correlation with TPP1, which functions as the principal route for POT1’s recruitment to telomeres. POT1 is known to disrupt the telomeric G-quadruplex (see below), enabling telomerase extension.^109^ However, it is believed that RAP1 in yeast binds to telomeric DNA to promote the synthesis of G-quadruplexes,^110^ whereas RAP1 in humans is recruited to telomeres by TRF2.^49^ It has been shown that TRF2 binds to T-loops and promotes their development (Figure 1).^25^ The shelterin complex as a whole controls signaling cascades from chromosomal ends and protects and regulates telomeres.^34^

Synthesis of the complementary C-strand by DNA polymerase α is also connected to telomere maintenance and cannot bind to ss telomeric DNA without the assistance of CTC1-STN1-TEN1 proteins known collectively as the ssDNA binding complex CST.^25,51,52^ Subunits of CST control access of telomerase, preventing G-overhang extension, while TEN1 is required for DNA polymerase α-mediated C-strand synthesis. In the cryo-EM structure (PDB ID 8SOK) with and without telomeric ssDNA, the CST complex can be observed to be interacting with POT1/TPP1 revealing how CST recruitment to the telomere is regulated by POT1 and its phosphorylation state.^51^

According to a recent study, human cells may include 200 telomere-associated proteins that interact with and may have an impact on telomeric structure.^111^ These were identified via the biochemical purification of the telomeric complexes. Given the wide variety of telomere components, it is possible that human telomeres are extremely malleable in their organization.

## High-Ordered Nucleic Acid Structures at the Telomeres

### G-Quadruplexes

Complementary DNA is associated with a double-stranded arrangement; however, it can also form multistranded structures beyond this duplex arrangement by either unzipping and then independently refolding of the two strands into alternative topologies or through the self-association of multiple strands.^81^ In regions containing telomeric DNA, the repetitive G-rich sequences can refold into a G-quadruplex (G4) (Figure 2B). Here the core structure consists of stacked G-quartets where the guanines associate with one another via Hoogsteen hydrogen bonding in a coplanar cyclic array, stabilized by eight hydrogen bonds. Through π–π stacking interactions, G-quartets can successfully stack on top of one another to create four-stranded G4 DNA structures.^112,113^ This arrangement results in a negatively charged central channel lined by carbonyl oxygen groups along the central axis of the structure. The charge repulsion in the channel is stabilized by the presence of monovalent cations. Each G4 consists of two distinct features: the centrally stacked G-stem and the unpaired bases that connect the guanine stretches to form the loops. The extended G-rich 3′ single-strand overhang at the ends of telomeres lacks the complementary C-rich strand and so provides an opportunity for these motifs to readily form and modulate access of POT1 to the ssDNA ends of the chromosomes.^32^ Structurally, it has been observed that G4s formed from human telomeric sequences can exist in multiple topologies.^114^ The several kb of single-stranded repeating G-rich sequences present at the telomeric ends can also form multiple G4 units adjacent to each other. These units can then combine to form complex multimeric G4 structures.^115−117^ The structure of the interface between dsDNA and G4 has been determined showing G4s stacked externally to the dsDNA providing a model for the 3′ end of a linear chromosome in the absence of POT1 (Figure 2C). The G4s are thermodynamically stable with melt temperature above dsDNA and can be further stabilized by the presence of small molecule ligands.^114^ It is worth mentioning that a G4 targeting clinical candidate—QN302—has been approved for Phase 1 clinical trials by the FDA for pancreatic ductal adenocarcinoma.^118,119^

Crucial to the discussion of G4 is the presence of G-quadruplex binding proteins (G4BPs) other than POT1 that perform several important functions like providing stability to the G4 complex as well as facilitating its unfolding.^120^ The basic categories of G4BPs are based on the regulatory mechanisms and functional interactions these proteins have with G4s. First, these proteins are classified as G4-folding proteins, which alter G4 structures, and G4-interacting proteins, which are functional proteins recruited by G4. Another way to categorize G4BPs is based on the distribution of G4s in the genome, i.e., DNA and RNA G4BPs.

G4BPs carry out a number of biological tasks like telomere homeostasis, which occurs at the site where telomere-binding proteins form a ternary complex with the G4 telomeric DNA structures.^66^ G4BPs such as helicases must resolve the G4s that arise during replication in order for the replication machinery to function properly. DNA2 is a helicase/nuclease protein that was initially identified in yeast but also isolated in mammalian DNA that localizes at telomeres and interacts with shelterin components TRF1 and TRF2. Using the helicase-dead DNA2 mutant protein, it was demonstrated that mammalian DNA2 nuclease identified and cleaved telomeric G4 DNA in a helicase-independent manner *in vitro*, leading to the nucleolytic elimination of both the G4 generated in 5′ flap structures and the telomeric G4 created in template DNA. The standard ssDNA repair apparatus could then probably close the resultant DNA gap in the template.^57^

The 5′–3′ DNA helicase FANCJ (Fanconi anemia complementation group J) is involved in a number of biological activities, including homologous recombination, DNA damage repair, G4 resolution, and maintaining genomic stability.^121^ In order to facilitate effective DNA replication, FANCJ may unfold and remove G4 structures; in contrast, lack of it will halt replication at G4s and ultimately result in DNA damage.^122^ It has been demonstrated that *S. cerevisiae’s* RecQ helicases Sgs1p and BLM preferentially unwind G4s over Holliday junctions. Researchers have also isolated a RecQ-like helicase called the StyRecQL, and it is evident that this helicase is linked to telomerase in the replication band, is drawn to replicating telomeres by telomerase, and plays a role in the unfolding of the G4.

TLS/FUS has also been identified that binds to G4 telomeric DNA and TERRA simultaneously. *In vitro*, a fold in the G4 Htelo and TERRA is the particular target of the C-terminal Arg-Gly-Gly (RGG) domain in TLS, which forms a ternary complex with them. Additionally, TLS binds G4 TERRA *in vivo* and G4 DNA in the telomere double-stranded region.^69^ Recently, researchers also isolated a nuclear protein that is highly prevalent in vertebrates called nuclear protein high mobility group B1 (HMGB1). HMGB1 shows a high affinity to bind to noncanonical DNA structures such as hemicatenated DNA loops and four-way junctions in addition to G4 DNA. Furthermore, it exhibits nonsequence selectivity in binding to B-form DNA, resulting in DNA helix deformation and promoting DNA interaction with other nuclear proteins.^71^

To aid in DNA replication, Pif1 helicase, another protein present in yeast cells, may attach to and unfold G4 structures.^60^ Pif1 prefers to attach itself to G4 forming sequences in the S-phase of the cell cycle.^61^ Pif1 helicase then unwinds any G4 structures, thereby reducing double strand breaks during the cell cycle.^123^ In the absence of Pif1, these sites are susceptible to double breaks.^124^ Furthermore, Pif1 is also negative regulator of telomerase.^62^

### T-Loop Structures

The main function of the long 3′ ssDNA overhang in mammalian telomeres may be to load POT1 and the shelterin complex at the end of the telomere, allowing them to interact with other shelterin complexes attached to other loops around the telomere.^25^ When the 3′ ssDNA overhang (TTAGGG in mammals) loops back and is tucked into the double-stranded component of the telomeric DNA molecule, lasso-like three-stranded DNA displacement loops known as T-loops or telomere loops are formed.^125^ These loops aid in concealing and protecting the single-strand overhangs of chromosomal DNA.^126^ According to a recently suggested T-loop concept,^125^ both terminal strands are annealed to their corresponding strands in the form of a bubble. Because this structural configuration possesses traits of both a replication fork and a Holliday junction, the T-loops are more stable within this configuration than with merely paired dsDNA with an ssDNA overhang. To further reinforce the stability of these T-loops, shelterin complexes loaded at the telomeric end preferentially loop back (Figure 1). As stated earlier, loading of POT1 and the shelterin complex at the terminal ends of the telomere allows them to interact with other shelterin complexes attached to different loops along the length of the telomere. This structural configuration, which is observed in naturally isolated T-loops, allows for a wider distribution of sizes for the circular part of the loop.^126^

**Figure 3:**
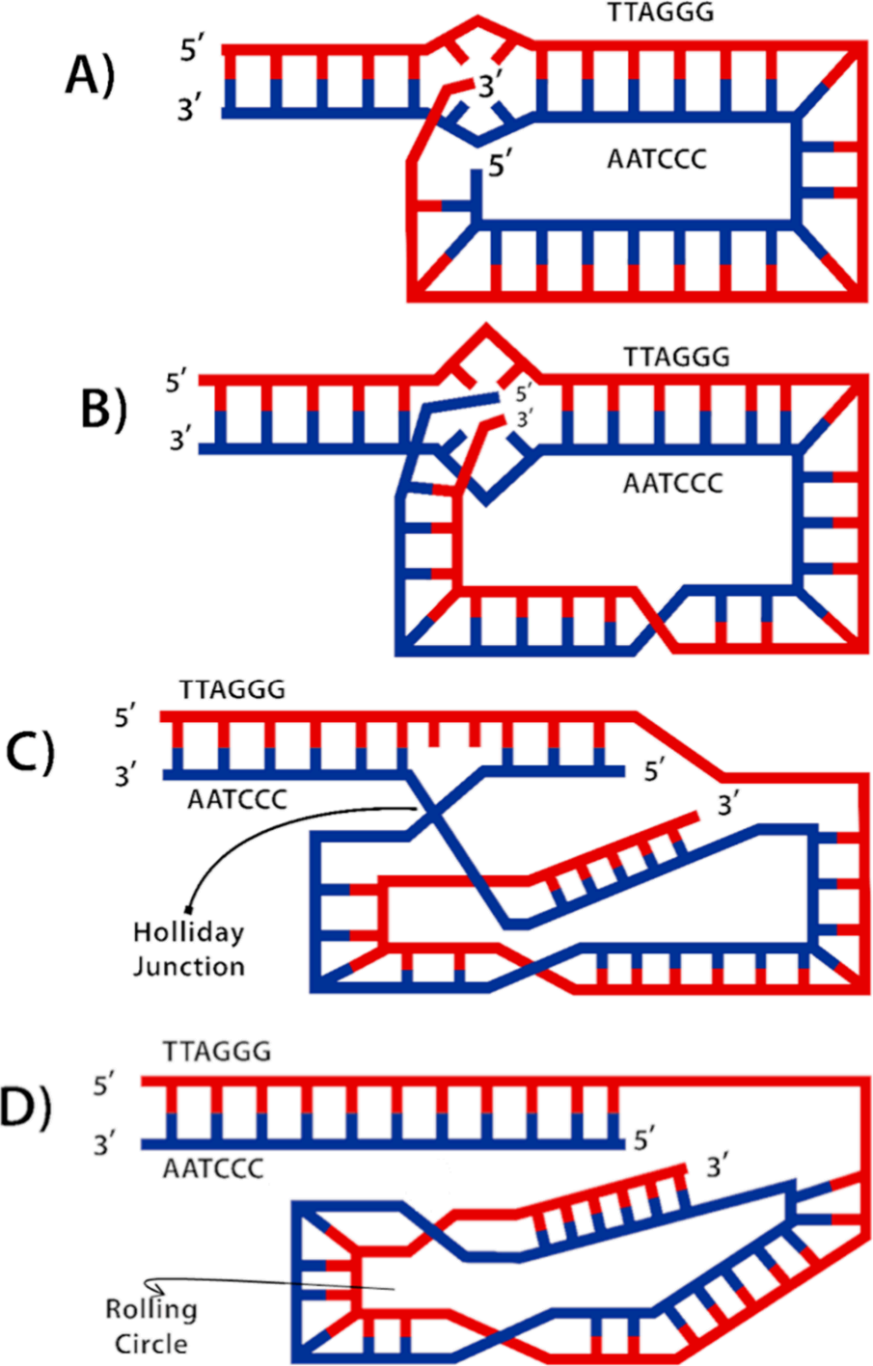
T-loop structures: (A) The integration of the 3′ terminal, which can serve as a replication origin, in both the structures depicted in A and B. Here, only the 3′ single-stranded overhang from the G-rich strand, annealed into the double-stranded DNA, is depicted in the conventional T-loop junction. (B) Terminal strands from a DNA molecule with blunt ends can be introduced to form a more stable T-loop structure. (C) Within the T-loop, an embedded replication origin and a Holliday junction can be observed. The presence of a classical Holliday junction is indicated by the topological equivalence between the structures in B and C. (D) Following resolution of the Holliday junction with resolvases like Gen1 or SLX1/4, a rolling circle replication template is generated.

The recently suggested model of the T-loop bubble states that the resolution of the Holliday junction (HJ) at the site where the two ssDNA cross over one another would result in a covalently closed ssDNA circle that anneals to the strand bearing the free 3′ terminus (Figure 3C,D).^126^ A rolling circular replication template that employs regular chromosomal replication components can significantly extend the preceding DNA by employing resolvases such as GEN1 or SLX1/4 to resolve the HJ at the T-loop. This theory offered an alternative mechanism by which the telomere may be extended by the T-loop. As was already established, the involvement of the T-loop in DNA transactions supports their function in telomere homeostasis. The origins of linear chromosomes and telomeres as well as how they overcome the difficulties associated with end-protection and end-replication, are still unknown. A recent study identified a potential CDK2 phosphorylation site at Ser365 in human TRF2, which may be mutated to alanine (Myc-tagged TRF2(S367A)) or treated with λ-protein phosphatase to eliminate it.^77^ This suggests a mechanism of unwinding the T-loops to facilitate telomere replication. The cell cycle analysis showed that this alteration is significantly less in the S phase but prevalent in the G1, G2, and M phases. This provides a brief window to the RTEL1 helicase in the S-phase during the PP6R3 phosphatase dephosphorylation to temporarily access and unwind T-loops and promote telomere replication.^77^

### D-Loops and Their Structures

During the formation of a D-loop, the dsDNA coils around in a lasso-like fashion.^47^ It is then possible for the single-stranded 3′ terminus overhang of telomeric DNA to re-enter the dsDNA and produce a displacement loop in the process (D-loop; Figure 3B).^81^ Once formed, D-loops are dynamic structures. A DNA polymerase that is committed to using donor DNA as a template during repair can expand D-loops with an annealed 3′-OH end. SGS1-TOP3-RMI1, MPH1, and SRS2 are proteins that dissolve D-loops by the use of helicases or topoisomerases.^80^ In the process of heteroduplex rejection, mismatch repair proteins improve the D-loop disruption mechanism as mismatched DNA. Sequence mismatches or differences could occur when DNA strands from different origins, such as sister chromatids or homologous chromosomes, construct a D-loop structure for repair or recombination. These discrepancies may result from DNA mistakes or genetic variances. By identification of these differences and mistakes within the D-loop, the mismatch repair proteins attach to the D-loop. The D-loop is then broken down, ensuring that only correctly matched DNA strands are used for repair or recombination, maintaining the integrity of the genetic material, and halting the spread of mutations.^127^ Due to the enzyme’s dynamic nature, two broken ends cannot invade the same donor molecule at the same time, resulting in the formation of a double-Holliday junction, or for a single end to invade two different donors at the same time, resulting in multi-invasions (MI). This prevents structure-selective endonucleases from modifying the donors in the covalent downstream covalent process.

D-loops are also important intermediaries during homologous recombination and a crucial step in the DNA double-strand break repair (DSBR) pathway.^84^ DSBR is a highly intricate process organized by a sophisticated interplay of enzymes and proteins. Key players in this regulatory network include ataxia-telangiectasia mutated (ATM) and ataxia-telangiectasia and RAD3 (ATR) related kinases, vital checkpoint enzymes finely attuned to detect DNA damage and activate the ensuing DNA damage response (DDR).^19^ ATM primarily addresses double-strand breakages, while ATR is specialized in responding to single-strand DNA damage. Both kinases phosphorylate downstream targets, instigating the initiation of repair processes.^85^ Another critical enzyme in this repair cascade is the MRN complex, comprising MRE11, RAD50, and NBS1 (also known as NBN).^88^ This multifaceted complex assumes a pivotal role in recognizing DSBs, processing DNA ends, and activating ATM. It serves to prime DNA ends for repair and is implicated in both nonhomologous end joining (NHEJ) and homologous recombination.^89^ RAD51, another essential enzyme, operates as a recombinase protein pivotal to homologous recombination (HR). It orchestrates the formation of nucleoprotein filaments on single-stranded DNA, facilitating the intricate process of strand exchange during HR.^92^

Beyond DSBR, several proteins, such as telomerase and shelterin complex components, rely on the structural properties of the telomeric DNA, including the D-loop, to carry out their functions.^81^ The D-loop within the telomeric DNA provides a structural feature that guides the telomerase to the appropriate location on the chromosome ends. It serves as a recognition site or a platform for telomerase to bind and accurately extend telomeric repeats. Without the D-loop, telomerase might have difficulty accessing and lengthening the telomeres effectively. The D-loop contributes to the proper assembly and stability of the shelterin complex. The D-loop provides structural cues that assist in the recruitment and positioning of shelterin proteins, ensuring the protection of telomeres from unwanted DNA damage responses.^25,81^

The length of the D-loops in human telomeric DNA may become longer, making it more difficult to break the D-loop. Therefore, it is possible that the DNA strand invasion machinery (which is by definition a propulsion mechanism for the route forward) is already inducing the reverse reaction by dictating the shape of the D-loop.^81^ If that is the case, it may be a component of the regulatory branch, which is responsible for promoting genome stability, and to prevent errors, maintain genomic integrity, safeguarding against mutations and diseases like cancer and act as a quality control system for DNA repair.^81,128^

### i-Loop Structures

**Figure 4:**
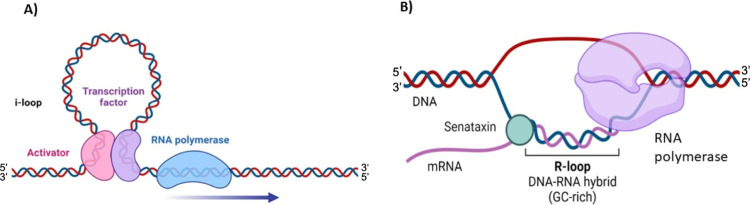
(A) DNA i-loop formation is a crucial step in gene transcription regulation. The DNA double helix serves as the backdrop, with RNA polymerase representing transcription initiation. Surrounding the DNA, transcription factors influence gene expression, and a specific activator protein (pink) triggers transcription in a precise DNA region. (B) The process of transcription with the formation and resolution of an R-loop is facilitated by the presence of RNA polymerase and the enzymatic action of senataxin. RNA polymerase initiates transcription by unwinding a section of the DNA, synthesizing a complementary RNA strand, and temporarily forming an R-loop as the nontemplate DNA strand is displaced and hybridized with the RNA. Senataxin (Sen1/SETX), depicted in purple, plays a critical role in resolving the R-loop, separating the DNA-RNA hybrid, allowing transcription to continue, and maintaining genomic stability. This process ensures the accurate synthesis of RNA transcripts and the proper functioning of transcription machinery.

Damage-induced loops are key intermediates (termed i-loops) that link telomere damage to telomere erosion and the generation of extrachromosomal telomeric t-circles (Figure 4A).^129,130^ The development of t-circles might be viewed as a result of chronic telomeric damage brought on by long-term chemotherapeutic actions that promote telomere shortening. Elements that prevent the exchange of chromosomal strands or cause inappropriate single-strand annealing at telomeres hinder the development of i-loops at the site of damage and eventually resist the development of extra-chromosomal t-circles. Additionally, damage to the telomeres would explain why many mutant genes involved in telomere maintenance have t-circles. Branch migration of i-loops, facilitated by specialized helicases such as RTEL1, BLM, and WRN, is self-induced when i-loops act as a substrate for these proteins at telomeric repeats.^130^ This process affects i-loop excision, which may reduce the probability of telomerase loss. Noncanonical telomeric repeats present in cells can also cause hindrance to telomerase loss in ALT (alternative lengthening of telomere) cells.^131^ Moreover, in yeast to humans, the development of circular DNAs may occur due to i-loops being produced from telomere damage, which occurred in other tandem repeats. Therefore, the i-loop rate would produce more repetitive elements with shorter repeated motifs due to the exposed complementary sequences after telomere damage. Extrachromosomal circles are most likely to be produced by telomeres with a repeat unit of 6 nt as compared to most other lengthy repeat units. The strong proclivity of telomeric repeats to form i-loops that may be excised as circles, resulting in continuous and random variations in the number of repeats, is one explanation for the diversity in the amplitude of telomere length across different chromosomes and cells.^130^

### R-Loop Structures

R-loops are unique nucleic acid structures generated when a newly transcribed RNA strand intrudes into the double-stranded DNA region following RNA polymerases, establishing an RNA-DNA hybrid (depicted in Figure 1). This process leads to the displacement of the nontemplate DNA strand, resulting in the formation of a single-stranded DNA (ssDNA) region. In the context of gene transcription, R-loops conventionally stem from regions rich in guanine clusters, also known as G-clusters (as illustrated in Figure 4B).^132^ Newly produced RNA is more likely to anneal with complementary ssDNA when these clusters are present.^133^ After the formation of the R-loop, stabilization and extension of the RNA-DNA hybridization are achieved, extended by the addition of successive guanine-rich (G-rich) regions. Elongation loses benefits as the structure breaks and the G-rich content decreases.^133^

The necessity for G-rich sequences is diminished, and R-loop formation is facilitated by additional variables. For example, interactions between the template strand and newly transcribed RNA are more likely when there is more negative supercoiling on the transcription bubble’s following fork. Moreover, even when the G-rich area is distant from the original G-cluster, nicks in the nontemplate strand can encourage DNA-RNA hybridization of developing RNA to the template strand.^133^ Among the many processes that contribute to maintaining the integrity of the transcription bubble and preventing R-loop accumulation are nuclease activity and topological stress reduction. These mechanisms are thoroughly described in recent review papers.^132−135^ To summarize, many enzymes work in tandem to prevent the accumulation of R-loops. RNase H1 and RNase H2 use 5′-3′ exonuclease activity to remove RNA from the loop. Since RNA-specific ribonucleases are the only enzymes that are known to break down hybridized RNA, these have been preserved throughout evolution in both prokaryotes and eukaryotes.^95^ In addition to RNase H1/2, cells include helicases like mammalian DHX9 and Aquarius that “untangle” DNA-RNA lesions (AQR). Two more helicases that are known to demolish R-loop structures are ATP-dependent DNA helicase senataxin and PIF1, an ortholog of the yeast Sen1p.^98^

**Figure 5:**
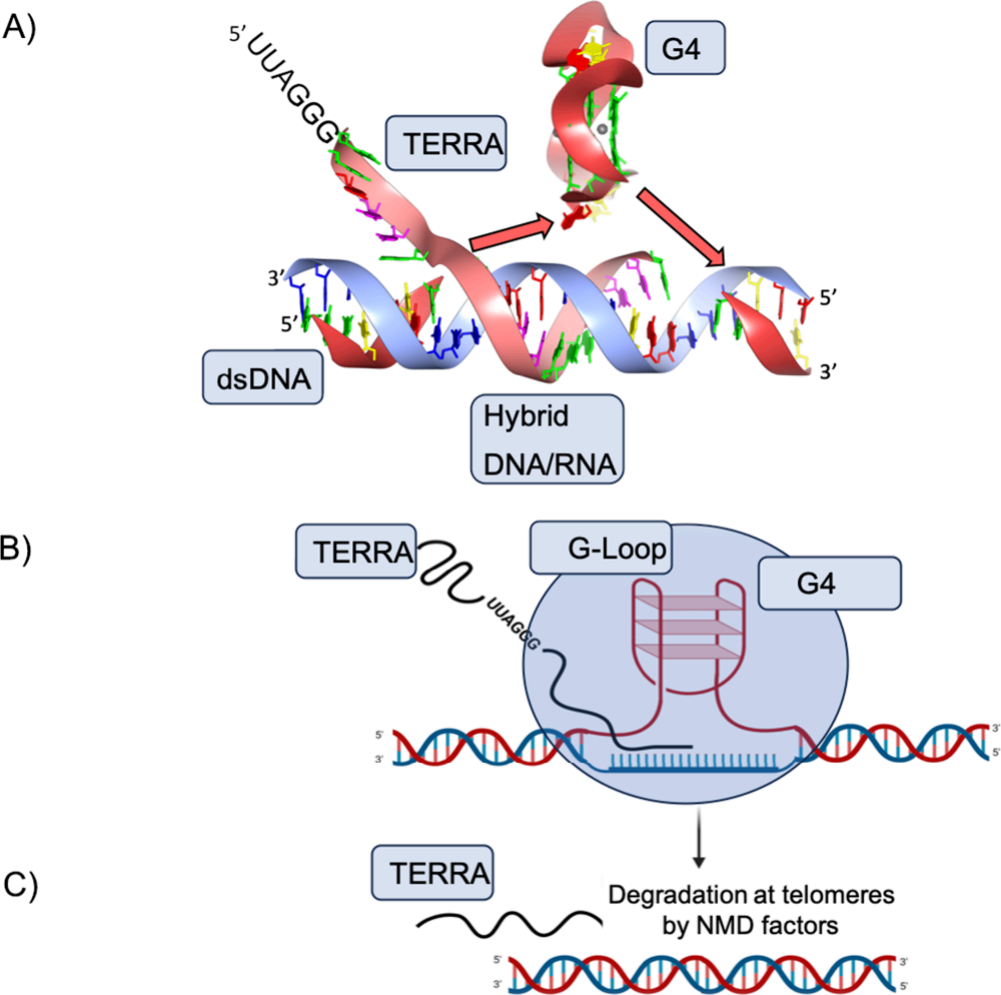
TERRA is a crucial component of telomeric heterochromatin, which also contains UUAGGG repeats formed from the transcription of telomeric regions and subtelomere-derived sequences. (A) A composite image highlighting known structural elements along with 3D spacial arrangements with the necessary rotational alignment and resulting topological complexity of these arrangements, R loop with insertion providing the framework for G4 formation. (B) The formation of the G-loop where the R-loop and G4 occur on opposing strands. (C) The NMD factors that interact with telomeric chromatin physically displace or degrade TERRA at telomeres.

Recently, structural motifs with cooperative relationships between G4s, TERRA (see below), and R-loops have been reported.^136^ These unique structures, termed G-loops, have been observed in ALT cells, where G4 and R-loop form on opposing strands^137^ (Figure 5B). The high G-loop levels in ALT cells suggest a plausible role these structural motifs play in the ALT maintenance mechanism. Moreover, the presence of G-loops is being presented as ALT biomarkers and potential therapeutic targets.^137^

### TERRA

Eukaryotic telomeres are transcribed into telomeric repeat-containing RNA (TERRA) despite the fact that they are heterochromatic structures.^4,18^ From several, if not all, chromosomal ends, TERRA molecules are translated in the direction of the telomeric DNA. They have UUAGGG repeats formed from transcription of telomeric regions and subtelomere-derived sequences (Figure 5A).^138^ It has been demonstrated that an increase in TERRA—the G-rich RNA—during the transcription of telomeres may also result in an increase in homologous recombination.^139^ Moreover, it has also been proposed that TERRA interferes with telomerase activity, which is responsible for lengthening the telomeric DNA at the ends of the chromosomes. Mammalian TERRA molecules have 5′-UUAGGG-3′ sequences that complement the hTR component of the telomerase enzyme’s template region.^104^ Since the TERRA and hTR components complement one another, it is possible that they will attach to telomerase directly and influence its activity. This theory is supported by the discovery that in cell extracts TERRA molecules are linked to human telomerase. Independent of hTR, researchers provided compelling evidence that TERRA molecules engage in interactions with hTERT.^104^

A cellular quality control system called NMD (nonsense-mediated decay) recognizes and breaks down aberrant RNA molecules with early stop codons, halting the synthesis of truncated or dysfunctional proteins.^103^ It helps ensure the accuracy and integrity of gene expression by eliminating potentially harmful or nonfunctional RNA transcripts. NMD factors are crucial in maintaining genomic stability, especially at telomeres. These factors physically interact with telomeric chromatin, the DNA–protein complex at telomeres. When they do so, two important actions take place regarding TERRA. First, NMD factors can displace TERRA from its telomeric position by competitively binding to it and in the process effectively removing it from the telomeric region. Second, NMD factors can contribute to the degradation of TERRA, thereby marking it for disposal when it is displaced or targeted by these factors (Figure 5B).^104^

In complex eukaryotes like humans, the recruitment and activation of telomerase at chromosomal ends is not well-known, and the function of EST1A (Ever Shorter Telomeres 1)/SMG6 in this process is unclear although human EST1A/SMG6 physically interacts with telomerase in a manner similar to yeast Est1.^104^ Though its effects on TERRA displacement at telomeres imply that EST1A/SMG6 may regulate telomerase through TERRA, its relationship with telomerase is consistent with a role in telomerase regulation.^18^ It has been hypothesized that TERRA may control telomerase in a telomere-length-dependent way, since TERRA is more prevalent when telomeres are long and the TERRA-mimicking RNA oligonucleotide (UUAGGGG)_3_ suppresses telomerase activity *in vitro* as evaluated by the TRAP assay.^140^ The discovery that some cancers had lower levels of TERRA than the equivalent normal tissue is also consistent with the idea that TERRA regulates telomerase negatively.^18,138^

By physically interacting with telomerase, hEST1A/SMG6 plays a significant function among the many proteins of the NMD process.^103^ Thus, it is possible to hypothesize that human EST1A/SMG6 may affect telomerase via TERRA regulation. Studies both *in vivo* and *in vitro* have demonstrated that TERRA at least partially controls the telomerase.^141^ Researchers have shown that there should be a sufficient equilibrium between TERRA formation, telomerase availability, RNA binding protein hnRNPA1, as well as free 3′ overhang of telomeric DNA at a specific period.^142^ When TERRA production exceeds hnRNPA1 abundance, hnRNPA1 can access the 3′ end of telomeric DNA and prevent telomerase from extending it, preventing it from binding to the telomeres.^18,138^

Direct correlations between TERRA and homologous recombinant (HR) factors such as RAD51, BRCA1, and RTEL have been shown in recent studies.^93,108,143,144^ It was demonstrated that TERRA stimulates R-loop formation at telomeres and starts strand invasion, which is reliant on RAD51. Similarly, RNA binding activity is shown in RAD51-associated protein 1 (RAD51AP1), a component that plays important roles in ALT pathways. Through specific HR intermediates known as DR-loops, RAD51AP1 uses RNA as part of a system that creates R-loops and displacement (D)-loops. The HR-driven DSB repair (HR-DSBR)18 factor BRCA1 (p220) also deals with ssDNA damage at the R-loop termination sites. A recent study has also shown the role of TERRA and RAD51API to the ALT pathways in RAD52 knockout cells by promoting telomeric R-loop formation that leads to G4 formation in telomeres.^106^ The dynamic telomeric R-loops generated by TERRA and RAD51AP1 activate the RAD52-independent ALT pathway, which in turn triggers G4 to orchestrate an R-to-D-loop transition at telomeres to drive break-induced replication of telomeres.^106^ These mechanisms are being widely studied for their role in cancer therapies.

### i-Motif

**Figure 6:**
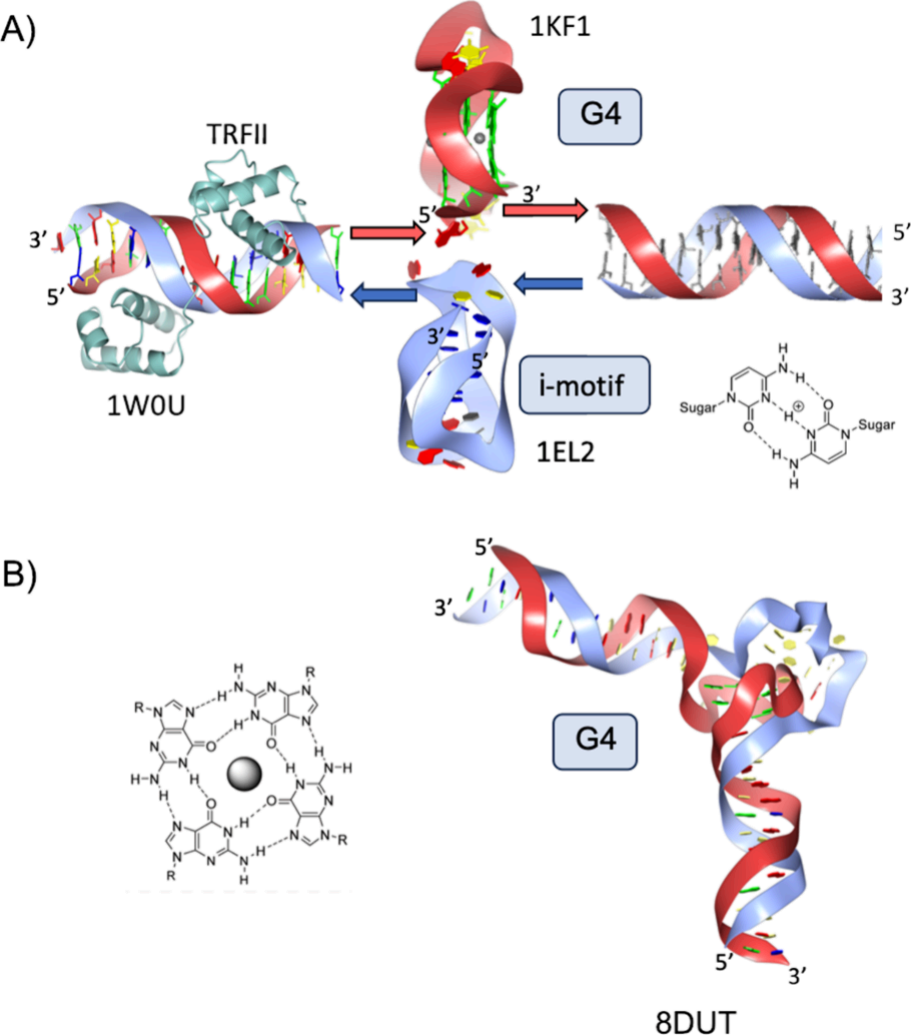
Models of motifs formed from human telomeric sequences. (A) Telomeric dsDNA bound by TRFII and TRFI (PDB 1W0U) adjacent to a bubble allowing the formation of G4 on the G-rich strand (PDB 1KF1) and an i-motif on the C-rich strand (PDB 1EL2). (B) The cryo-EM model of a G4 within the context of an open bubble containing a central G4 was determined (PDB 8DUT). Red arrows show the G-rich DNA direction, and blue arrows show the C-rich DNA 5′-3′ direction. A hemiprotonated cytosine-cytosine (C–C+) base pair and a G-tetrad have also been shown to highlight the hydrogen bonding patterns. The corresponding PDB identifiers are also listed below each subfigure.

i-Motifs are four-stranded DNA structures that are formed in sequences rich in cytosines in the adjacent unpaired DNA strand, just like the G-rich sequences form G4s.^145^ The two parallel stranded duplexes associate in a head-to-tail orientation within the i-motif upon the intercalation of the CC+ base pair (Figure 6A).^146^ At low pH values, this structure is stabilized by the protonation of cytosines. It has been proposed that this structure may affect the dynamics of the telomeric DNA duplex and promote its opening.^147^ The i-motifs may be classified into two main intercalated topologies: the 3′-E topology, where the outermost base pair (C–C) is located at the 3′-end, and the 5′-E topology, where the outermost base pair (C–C) is located at the 5′-end. Between these two topologies, the 3′-E topology is also more stable.^148^ The interactions between sugar–sugar contacts along the tiny grooves, which encourage optimal backbone twisting and the formation of stacking bases, are responsible for the molecule’s stability.^149^ The overall stability of i-motif structures, however, is determined by the number of cytosine residues that interact with one another. This suggests that when more cytosine residues form hydrogen bonds, the molecule will be more stable. In addition, other factors that affect the stability of the i-motif include temperature, salt content, and environmental pH.^150^ A great deal of research has been conducted to understand how i-motif structures behave in various environments. This includes examining the effects of altering the lengths of the cytosine tract and loop, utilizing cytosine analogs that have been epigenetically modified, and changing the DNA backbone as well as other modifications.^151^ Since pH plays a crucial part in regulating folding, its impact on the i-motif has been well studied, including how stable it is at various pH levels and how it affects the structure’s kinetic and thermodynamic characteristics.^152−154^ It should be noted that the thermodynamic and thermal stability are related but not identical properties of i-motifs.^155^ Conversely, the impact of the temperature on the structure has received far less attention. Unusual effects related to temperature have been previously noted in the i-motif while investigating its pH-responsiveness.^148,156,157^ This includes the isothermal hysteresis in pH transitions^158^ and the hysteresis that is frequently seen between thermal melting and annealing curves for the structure.^159^ It has also been discovered that kinetic partitioning occurs when a pH drop causes the i-motif to fold quickly into one conformation at first, but over time, it unfolds and refolds to a slower-forming, more stable conformation. As a result, the i-motif structure was described as residing in an equilibrium at a specific pH and temperature where conformers were slowly interconverting.^159^ The influence of the temperature is a significant variable that warrants careful examination because of its dynamic character.

The most stable pH range for human telomeric i-motif complexes is ∼6.0.^159^ The use of a free proton by the nucleic acids during the folding process has been found to allow certain i-motifs to form at neutral pH.^148^ To detect the i-motif complexes, certain parameters must be met, including molecular crowding, negative superhelicity, and a temperature of 4 °C.^160^ Furthermore, for i-motifs to be stable at a neutral pH, the superhelicity must remain negative.^161^

## Holliday Junctions and Telomeres

**Figure 7:**
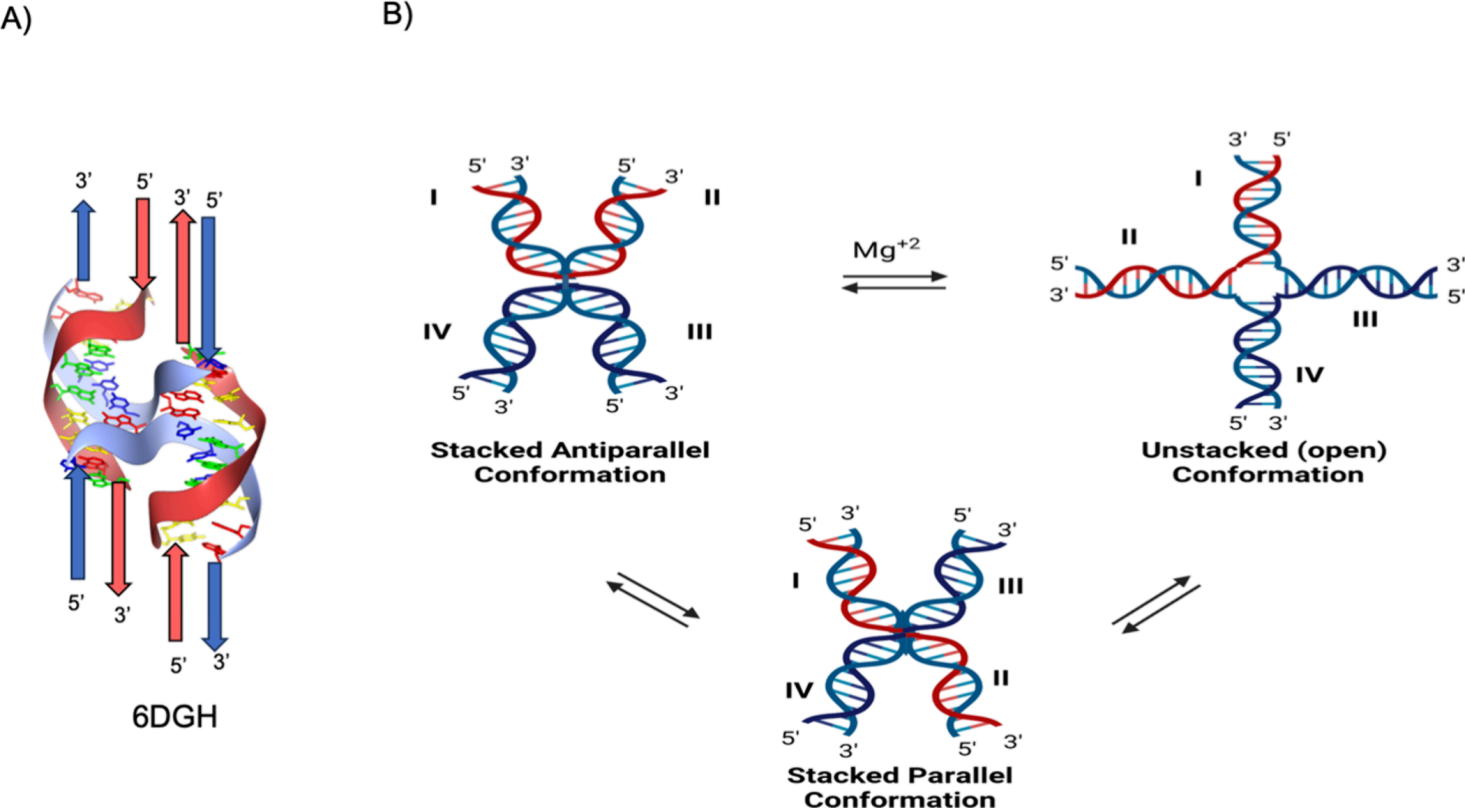
Potential Holliday junction conformations. (A) Crystal structure of a single Holliday junction formed from telomeric DNA folded in the stacked X-conformation (PDB 6DGH). Strand exchange occurs via the C-rich strand. Red arrows depict the G-rich strand; blue arrows depict the C-rich strand. (B) Three conformations of a Holliday junction parallel, open, and stacked-X antiparallel HJs. In the presence of magnesium ions, the HJ formation undergoes a transition from an open to a stacked antiparallel topological state.

Holliday junctions (HJ) reflect branched nucleic acid structures that consist of two pairs of double-stranded arms joined together.^162^ HJs are intermediates of homologous recombination (HR).^163^ HR is critical during meiosis because it promotes genetic variety by allowing the flow of genetic material across cells. HJs form a covalent bond between DNA molecules that are undergoing recombination during mitosis, and as a result, they must be removed before the chromosomes are segregated.^164^ The inability of the HJ to resolve results in severe mitotic repercussions resulting in the creation of DNA breaks and chromosomal abnormalities.^165^ Although high-density lipoproteins are produced to aid in the effectiveness of DNA repair, they are also believed to be toxic, because they have the ability to interfere with proper chromosome segregation (chromosome separation). The primary aim of the HJ is to allow the exchange of distinct portions of genetic information. Three structural arrangements of telomeric HJs have been reported including single, double, and protein-associated HJs (Figure 7A).^166^

*In vivo*, HJ exhibits different structural variants. When the HJ is free in solution, it acquires a variety of different interconvertible configurations (Figure 7B).^167^ The junction expands to an open form in the presence of low salt conditions and absence of multivalent ions, which lessens the repulsion between the negatively charged phosphates concentrated at the junction.^166,168^ When either a large concentration of monovalent cations or a high concentration of multivalent cations is present, the junction overcomes electrostatic repulsion and folds into one of two stacked conformers.^167,168^ Although HJs are produced to aid in the effectiveness of DNA repair, they are also regarded to be toxic because they have the potential to interfere with normal chromosome segregation.^169^ The ability to recognize distinct physical and geometric features of the junctions is necessary to comprehend how the HJ is processed by proteins.

Single HJs represent structures that occur independently. In mitotic cells, single HJs are resolved by structure-selective endonucleases known as HJ resolvases.^170^ This type of junction typically consists of a symmetrical sequence that allows them to move freely, which means that the four single arms can slide in a particular pattern through the junction depending on the base-pairing.^171^ The main goal of single HJs is to facilitate the repair of the breaks encountered in double strands. A study initiated by Haider et al. revealed that the C-rich lagging strand contains structural features that constrain crossover geometry and allow the formation of the telomeric HJs.^166^

**Figure 8:**
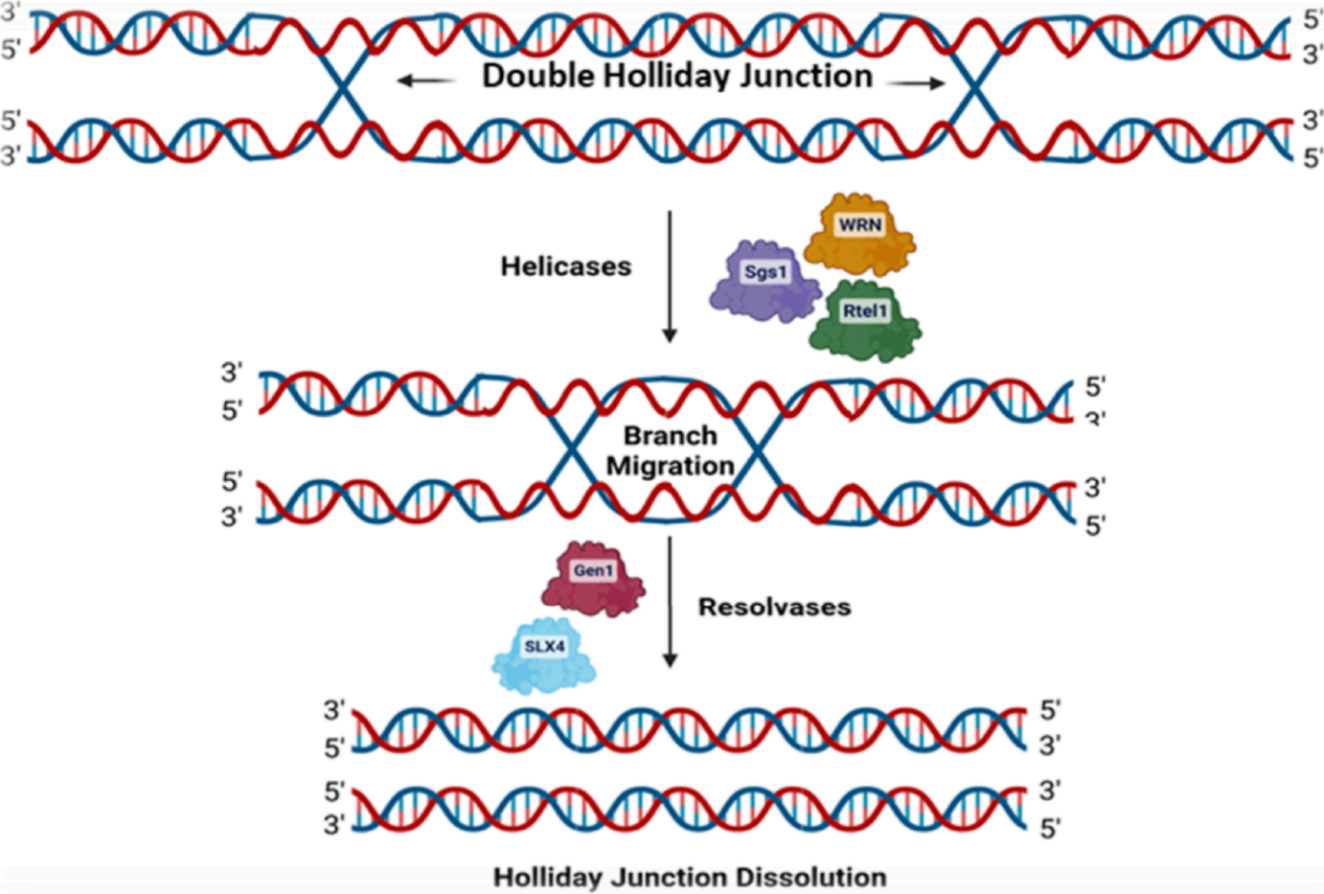
Regulation of double HJ resolution. Several helicase molecules can bring double-Holliday junctions very near together, and then, the Holliday junction can be resolved by resolvases such as Gen1 or SLX1/4.

When two single HJs are topologically linked within shared proximity, they are defined as double-Holliday junctions (dHJ; Figure 8). Like single HJs, dHJs also reflect the critical intermediates of the homologous recombination process.^172^ The components consist of separate links that can be cleaved via DNA structure-selective endonucleases, popularly referred to as HJ resolvases.^173^ In other instances, dHJ can undergo processing via a reaction known as “the dissolution of double-Holliday junction”, which requires the cooperative action of several enzymes.^172^ dHJs play the critical role of ensuring the migration of different HJs toward each other to establish a hemicatenated intermediate, which can undergo decatenation via topoisomerase during the dissolution procedure.^172^

At the telomeres, the dHJ helps in restructuring the telomeric proteins within the cells so that the potential chance of cancer can be reduced. Single and double HJs are key intermediates of the ALT pathway.^174^ This recombination-mediated telomere maintenance method progresses via the formation of HJ intermediates and is central to telomerase-negative maintenance of telomeres.^174^

## Conclusion

The ends of chromosomes of eukaryotes are preserved by the telomere, which are structurally composed of nucleoprotein complexes. The telomere is especially exposed to continuous shrinkage as the DNA replicates during the continuous regeneration of tissue, thereby conferring a high risk of chromosomal instability. Telomeric erosion has been observed in aging tissues and hyperproliferative disease states—both of which are associated with an elevated risk of cancer. Telomere protection failure can result in either degenerative aging or malignancy, with the specific outcome determined by the integrity of DNA damage checkpoint responses. It is important to note that the reactivation of telomerase helps to preserve telomere length in many of the advanced cancers, such as epithelial cancers. Numerous regulatory pathways for telomere length regulation have been reported, and genome-scale investigations have aided in the identification of genes involved in telomere length management. These observations underpin the premise that the degradation of telomeres identified in aging people may support the various aging phenotypes. Consequently, a degraded telomere is a representation of a significant genotoxic indicator, which can trigger DNA damage indicating pathways with the ability to speed up aging. It is essential to secure the chromosome ends from the reaction to the triggers due to damage of DNA. The mending of DNA pathways is accomplished by the activity of particular proteins that produce telomeres at the ends of chromosomes. Telomeres must be regulated and maintained because they are heterochromatic and fold into certain configurations (T-loops), or with the formation of G4s at the 3′ ssDNA ends, which might obstruct DNA replication. Telomeres with altered shape or chromosome ends that are severely short generate defective telomeres and eventually result in replicative senescence or chromosome instability. Emerging data suggest that TERRA, a type of long noncoding RNA transcribed at telomeres, is involved in the mechanisms governing telomere preservation and chromosomal end-protection.

## References

[ref1] Smith E. M.; Pendlebury D. F.; Nandakumar J. Structural biology of telomeres and telomerase. Cell. Mol. Life Sci. 2020, 77, 61–79. 10.1007/s00018-019-03369-x. 31728577 PMC6986361

[ref2] Shay J. W.; Wright W. E. Telomeres and telomerase: three decades of progress. Nat. Rev. Genet. 2019, 20, 299–309. 10.1038/s41576-019-0099-1. 30760854

[ref3] Sobinoff A. P.; Pickett H. A. Mechanisms that drive telomere maintenance and recombination in human cancers. Curr. Opin. Genet. Dev. 2020, 60, 25–30. 10.1016/j.gde.2020.02.006. 32119936

[ref4] Cusanelli E.; Chartrand P. Telomeric repeat-containing RNA TERRA: a noncoding RNA connecting telomere biology to genome integrity. Front. Genet. 2015, 6, 143 10.3389/fgene.2015.00143. 25926849 PMC4396414

[ref5] Koneru B.; Lopez G.; Farooqi A.; Conkrite K. L.; Nguyen T. H.; Macha S. J.; Modi A.; Rokita J. L.; Urias E.; Hindle A.; Davidson H.; Mccoy K.; Nance J.; Yazdani V.; Irwin M. S.; Yang S.; Wheeler D. A.; Maris J. M.; Diskin S. J.; Reynolds C. P. Telomere Maintenance Mechanisms Define Clinical Outcome in High-Risk Neuroblastoma. Cancer Res. 2020, 80, 2663–2675. 10.1158/0008-5472.CAN-19-3068. 32291317 PMC7313726

[ref6] Zhu H.; Belcher M.; van der Harst P. Healthy aging and disease: role for telomere biology?. Clin. Sci. 2011, 120, 427–440. 10.1042/CS20100385. PMC3035527 21271986

[ref7] Zakian V. A. Telomeres: The beginnings and ends of eukaryotic chromosomes. Exp. Cell Res. 2012, 318, 1456–1460. 10.1016/j.yexcr.2012.02.015. 22391099 PMC3372703

[ref8] Oeseburg H.; De Boer R. A.; Van Gilst W. H.; Van Der Harst P. Telomere biology in healthy aging and disease. Pflüg. Arch. - Eur. J. Physiol. 2010, 459, 259–268. 10.1007/s00424-009-0728-1. PMC2801851 19756717

[ref9] Srinivas N.; Rachakonda S.; Kumar R. Telomeres and Telomere Length: A General Overview. Cancers 2020, 12, 558 10.3390/cancers12030558. 32121056 PMC7139734

[ref10] Wright W. E.; Tesmer V. M.; Huffman K. E.; Levene S. D.; Shay J. W. Normal human chromosomes have long G-rich telomeric overhangs at one end. Genes Dev. 1997, 11, 2801–2809. 10.1101/gad.11.21.2801. 9353250 PMC316649

[ref11] Hwang H.; Kreig A.; Calvert J.; Lormand J.; Kwon Y.; Daley J. M.; Sung P.; Opresko P. L.; Myong S. Telomeric Overhang Length Determines Structural Dynamics and Accessibility to Telomerase and ALT-Associated Proteins. Structure 2014, 22, 842–853. 10.1016/j.str.2014.03.013. 24836024 PMC4067947

[ref12] Jafri M. A.; Ansari S. A.; Alqahtani M. H.; Shay J. W. Roles of telomeres and telomerase in cancer, and advances in telomerase-targeted therapies. Genome Med. 2016, 8, 69 10.1186/s13073-016-0324-x. 27323951 PMC4915101

[ref13] Nabetani A.; Ishikawa F. Unusual Telomeric DNAs in Human Telomerase-Negative Immortalized Cells. Mol. Cell. Biol. 2009, 29, 703–713. 10.1128/MCB.00603-08. 19015236 PMC2630689

[ref14] Greider C. W. Regulating telomere length from the inside out: the replication fork model. Genes Dev. 2016, 30, 1483–1491. 10.1101/gad.280578.116. 27401551 PMC4949321

[ref15] Siderakis M.; Tarsounas M. Telomere regulation and function during meiosis. Chromosome Res. 2007, 15, 667–679. 10.1007/s10577-007-1149-7. 17674153 PMC2459255

[ref16] Lee K.-H.; Kim D.-Y.; Kim W. Regulation of Gene Expression by Telomere Position Effect. Int. J. Mol. Sci. 2021, 22, 12807 10.3390/ijms222312807. 34884608 PMC8657463

[ref17] Martínez P.; Blasco M. A. Telomeric and extra-telomeric roles for telomerase and the telomere-binding proteins. Nat. Rev. Cancer 2011, 11, 161–176. 10.1038/nrc3025. 21346783

[ref18] Luke B.; Lingner J. TERRA: telomeric repeat-containing RNA. EMBO J. 2009, 28, 2503–2510. 10.1038/emboj.2009.166. 19629047 PMC2722245

[ref19] Lue N. F.; Yu E. Y. Telomere recombination pathways: tales of several unhappy marriages. Curr. Genet. 2017, 63, 401–409. 10.1007/s00294-016-0653-8. 27666406 PMC5366096

[ref20] Pardue M.-L.; DeBaryshe P. G. Retrotransposons that maintain chromosome ends. Proc. Natl. Acad. Sci. U. S. A. 2011, 108, 20317–20324. 10.1073/pnas.1100278108. 21821789 PMC3251079

[ref21] Rubtsova M.; Dontsova O. Human Telomerase RNA: Telomerase Component or More?. Biomolecules 2020, 10, 873 10.3390/biom10060873. 32517215 PMC7355840

[ref22] Galati A.; Scatolini L.; Micheli E.; Bavasso F.; Cicconi A.; Maccallini P.; Chen L.; Roake C. M.; Schoeftner S.; Artandi S. E.; Gatti M.; Cacchione S.; Raffa G. D. The S-adenosylmethionine analog sinefungin inhibits the trimethylguanosine synthase TGS1 to promote telomerase activity and telomere lengthening. FEBS Lett. 2022, 596, 42–52. 10.1002/1873-3468.14240. 34817067

[ref23] Buseman C. M.; Wright W. E.; Shay J. W. Is telomerase a viable target in cancer?. Mutat. Res. Mol. Mech. Mutagen. 2012, 730, 90–97. 10.1016/j.mrfmmm.2011.07.006. PMC3375693 21802433

[ref24] Ghanim G. E.; Fountain A. J.; Van Roon A.-M. M.; Rangan R.; Das R.; Collins K.; Nguyen T. H. D. Structure of human telomerase holoenzyme with bound telomeric DNA. Nature 2021, 593, 449–453. 10.1038/s41586-021-03415-4. 33883742 PMC7610991

[ref25] Lim C. J.; Cech T. R. Shaping human telomeres: from shelterin and CST complexes to telomeric chromatin organization. Nat. Rev. Mol. Cell Biol. 2021, 22, 283–298. 10.1038/s41580-021-00328-y. 33564154 PMC8221230

[ref26] Liu B.; He Y.; Wang Y.; Song H.; Zhou Z. H.; Feigon J. Structure of active human telomerase with telomere shelterin protein TPP1. Nature 2022, 604, 578–583. 10.1038/s41586-022-04582-8. 35418675 PMC9912816

[ref27] Sekne Z.; Ghanim G. E.; Van Roon A.-M. M.; Nguyen T. H. D. Structural basis of human telomerase recruitment by TPP1-POT1. Science 2022, 375, 1173–1176. 10.1126/science.abn6840. 35201900 PMC7612489

[ref28] Zinder J. C.; Olinares P. D. B.; Svetlov V.; Bush M. W.; Nudler E.; Chait B. T.; Walz T.; De Lange T. Shelterin is a dimeric complex with extensive structural heterogeneity. Proc. Natl. Acad. Sci. U. S. A. 2022, 119, e2201662119 10.1073/pnas.2201662119. 35881804 PMC9351484

[ref29] Lin J.; Countryman P.; Buncher N.; Kaur P.; E L.; Zhang Y.; Gibson G.; You C.; Watkins S. C.; Piehler J.; Opresko P. L.; Kad N. M.; Wang H. TRF1 and TRF2 use different mechanisms to find telomeric DNA but share a novel mechanism to search for protein partners at telomeres. Nucleic Acids Res. 2014, 42, 2493–2504. 10.1093/nar/gkt1132. 24271387 PMC3936710

[ref30] Lei M.; Podell E. R.; Baumann P.; Cech T. R. DNA self-recognition in the structure of Pot1 bound to telomeric single-stranded DNA. Nature 2003, 426, 198–203. 10.1038/nature02092. 14614509

[ref31] Linger B. R.; Morin G. B.; Price C. M. The Pot1a-associated proteins Tpt1 and Pat1 coordinate telomere protection and length regulation in Tetrahymena. Mol. Biol. Cell 2011, 22, 4161–4170. 10.1091/mbc.e11-06-0551. 21900503 PMC3204076

[ref32] Tesmer V. M.; Brenner K. A.; Nandakumar J. Human POT1 protects the telomeric ds-ss DNA junction by capping the 5′ end of the chromosome. Science 2023, 381, 771–778. 10.1126/science.adi2436. 37590346 PMC10666826

[ref33] Aramburu T.; Kelich J.; Rice C.; Skordalakes E. POT1-TPP1 binding stabilizes POT1, promoting efficient telomere maintenance. Comput. Struct. Biotechnol. J. 2022, 20, 675–684. 10.1016/j.csbj.2022.01.005. 35140887 PMC8803944

[ref34] Ghilain C.; Gilson E.; Giraud-Panis M.-J. Multifunctionality of the Telomere-Capping Shelterin Complex Explained by Variations in Its Protein Composition. Cells 2021, 10, 1753 10.3390/cells10071753. 34359923 PMC8305809

[ref35] Xin H.; Liu D.; Songyang Z. The telosome/shelterin complex and its functions. Genome Biol. 2008, 9, 232 10.1186/gb-2008-9-9-232. 18828880 PMC2592706

[ref36] Grill S.; Nandakumar J. Molecular mechanisms of telomere biology disorders. J. Biol. Chem. 2021, 296, 100064 10.1074/jbc.REV120.014017. 33482595 PMC7948428

[ref37] Hu C.; Rai R.; Huang C.; Broton C.; Long J.; Xu Y.; Xue J.; Lei M.; Chang S.; Chen Y. Structural and functional analyses of the mammalian TIN2-TPP1-TRF2 telomeric complex. Cell Res. 2017, 27, 1485–1502. 10.1038/cr.2017.144. 29160297 PMC5717407

[ref38] Wang F.; Podell E. R.; Zaug A. J.; Yang Y.; Baciu P.; Cech T. R.; Lei M. The POT1–TPP1 telomere complex is a telomerase processivity factor. Nature 2007, 445, 506–510. 10.1038/nature05454. 17237768

[ref39] Glousker G.; Briod A.; Quadroni M.; Lingner J. Human shelterin protein POT1 prevents severe telomere instability induced by homology-directed DNA repair. EMBO J. 2020, 39, e104500 10.15252/embj.2020104500. 33073402 PMC7705456

[ref40] Diotti R.; Loayza D. Shelterin complex and associated factors at human telomeres. Nucleus 2011, 2, 119–135. 10.4161/nucl.2.2.15135. 21738835 PMC3127094

[ref41] De Lange T. How Shelterin Solves the Telomere End-Protection Problem. Cold Spring Harb. Symp. Quant. Biol. 2010, 75, 167–177. 10.1101/sqb.2010.75.017. 21209389

[ref42] Hu H.; Van Roon A.-M. M.; Ghanim G. E.; Ahsan B.; Oluwole A. O.; Peak-Chew S.-Y.; Robinson C. V.; Nguyen T. H. D. Structural basis of telomeric nucleosome recognition by shelterin factor TRF1. Sci. Adv. 2023, 9, eadi4148 10.1126/sciadv.adi4148. 37624885 PMC10456876

[ref43] Court R.; Chapman L.; Fairall L.; Rhodes D. How the human telomeric proteins TRF1 and TRF2 recognize telomeric DNA: a view from high-resolution crystal structures. EMBO Rep. 2005, 6, 39–45. 10.1038/sj.embor.7400314. 15608617 PMC1299224

[ref44] Jones M.; Bisht K.; Savage S. A.; Nandakumar J.; Keegan C. E.; Maillard I. The shelterin complex and hematopoiesis. J. Clin. Invest. 2016, 126, 1621–1629. 10.1172/JCI84547. 27135879 PMC4855927

[ref45] Kim S.; Kaminker P.; Campisi J. TIN2, a new regulator of telomere length in human cells. Nat. Genet. 1999, 23, 405–412. 10.1038/70508. 10581025 PMC4940194

[ref46] Pike A. M.; Strong M. A.; Ouyang J. P. T.; Greider C. W. TIN2 Functions with TPP1/POT1 To Stimulate Telomerase Processivity. Mol. Cell. Biol. 2019, 39, e00593-18 10.1128/MCB.00593-18. 31383750 PMC6791651

[ref47] Rai R.; Chen Y.; Lei M.; Chang S. TRF2-RAP1 is required to protect telomeres from engaging in homologous recombination-mediated deletions and fusions. Nat. Commun. 2016, 7, 10881 10.1038/ncomms10881. 26941064 PMC4785230

[ref48] Lototska L.; Yue J.; Li J.; Giraud-Panis M.; Songyang Z.; Royle N. J.; Liti G.; Ye J.; Gilson E.; Mendez-Bermudez A. Human RAP1 specifically protects telomeres of senescent cells from DNA damage. EMBO Rep. 2020, 21, e49076 10.15252/embr.201949076. 32096305 PMC7132343

[ref49] Janoušková E.; Nečasová I.; Pavloušková J.; Zimmermann M.; Hluchý M.; Marini V.; Nováková M.; Hofr C. Human Rap1 modulates TRF2 attraction to telomeric DNA. Nucleic Acids Res. 2015, 43, 2691–2700. 10.1093/nar/gkv097. 25675958 PMC4357705

[ref50] Chen Y.; Rai R.; Zhou Z.-R.; Kanoh J.; Ribeyre C.; Yang Y.; Zheng H.; Damay P.; Wang F.; Tsujii H.; Hiraoka Y.; Shore D.; Hu H.-Y.; Chang S.; Lei M. A conserved motif within RAP1 has diversified roles in telomere protection and regulation in different organisms. Nat. Struct. Mol. Biol. 2011, 18, 213–221. 10.1038/nsmb.1974. 21217703 PMC3688267

[ref51] Cai S. W.; Takai H.; Walz T.; De Lange T. Structural basis of CST-Polα/Primase recruitment and regulation by POT1 at telomeres. bioRxiv. 2023.

[ref52] He Q.; Lin X.; Chavez B. L.; Agrawal S.; Lusk B. L.; Lim C. J. Structures of the human CST-Polα–primase complex bound to telomere templates. Nature 2022, 608, 826–832. 10.1038/s41586-022-05040-1. 35830881 PMC10268231

[ref53] Miyake Y.; Nakamura M.; Nabetani A.; Shimamura S.; Tamura M.; Yonehara S.; Saito M.; Ishikawa F. RPA-like mammalian Ctc1-Stn1-Ten1 complex binds to single-stranded DNA and protects telomeres independently of the Pot1 pathway. Mol. Cell 2009, 36, 193–206. 10.1016/j.molcel.2009.08.009. 19854130

[ref54] Lim C. J.; Barbour A. T.; Zaug A. J.; Goodrich K. J.; McKay A. E.; Wuttke D. S.; Cech T. R. The structure of human CST reveals a decameric assembly bound to telomeric DNA. Science 2020, 368, 1081–1085. 10.1126/science.aaz9649. 32499435 PMC7559292

[ref55] Bryan C.; Rice C.; Harkisheimer M.; Schultz D. C.; Skordalakes E. Structure of the Human Telomeric Stn1-Ten1 Capping Complex. PLoS One 2013, 8, e66756 10.1371/journal.pone.0066756. 23826127 PMC3691326

[ref56] Grandin N. Ten1 functions in telomere end protection and length regulation in association with Stn1 and Cdc13. EMBO J. 2001, 20, 1173–1183. 10.1093/emboj/20.5.1173. 11230140 PMC145504

[ref57] Lin W.; Sampathi S.; Dai H.; Liu C.; Zhou M.; Hu J.; Huang Q.; Campbell J.; Shin-Ya K.; Zheng L.; Chai W.; Shen B. Mammalian DNA2 helicase/nuclease cleaves G-quadruplex DNA and is required for telomere integrity. EMBO J. 2013, 32, 1425–1439. 10.1038/emboj.2013.88. 23604072 PMC3655473

[ref58] Zheng L.; Meng Y.; Campbell J. L.; Shen B. Multiple roles of DNA2 nuclease/helicase in DNA metabolism, genome stability and human diseases. Nucleic Acids Res. 2020, 48, 16–35. 10.1093/nar/gkz1101. 31754720 PMC6943134

[ref59] Zhou C.; Pourmal S.; Pavletich N. P. Dna2 nuclease-helicase structure, mechanism and regulation by Rpa. eLife 2015, 4, e09832 10.7554/eLife.09832. 26491943 PMC4716839

[ref60] Byrd A. K.; Bell M. R.; Raney K. D. Pif1 helicase unfolding of G-quadruplex DNA is highly dependent on sequence and reaction conditions. J. Biol. Chem. 2018, 293, 17792–17802. 10.1074/jbc.RA118.004499. 30257865 PMC6240867

[ref61] Paeschke K.; Capra J. A.; Zakian V. A. DNA Replication through G-Quadruplex Motifs Is Promoted by the Saccharomyces cerevisiae Pif1 DNA Helicase. Cell 2011, 145, 678–691. 10.1016/j.cell.2011.04.015. 21620135 PMC3129610

[ref62] Schulz V. P.; Zakian V. A. The saccharomyces PIF1 DNA helicase inhibits telomere elongation and de novo telomere formation. Cell 1994, 76, 145–155. 10.1016/0092-8674(94)90179-1. 8287473

[ref63] Dehghani-Tafti S.; Levdikov V.; Antson A. A.; Bax B.; Sanders C. M. Structural and functional analysis of the nucleotide and DNA binding activities of the human PIF1 helicase. Nucleic Acids Res. 2019, 47, 3208–3222. 10.1093/nar/gkz028. 30698796 PMC6451128

[ref64] Su N.; Byrd A. K.; Bharath S. R.; Yang O.; Jia Y.; Tang X.; Ha T.; Raney K. D.; Song H. Structural basis for DNA unwinding at forked dsDNA by two coordinating Pif1 helicases. Nat. Commun. 2019, 10, 5375 10.1038/s41467-019-13414-9. 31772234 PMC6879534

[ref65] Dai Y.; Guo H.; Liu N.; Chen W.; Ai X.; Li H.; Sun B.; Hou X.; Rety S.; Xi X. Structural mechanism underpinning Thermus oshimai Pif1-mediated G-quadruplex unfolding. EMBO Rep. 2022, 23, e53874 10.15252/embr.202153874. 35736675 PMC9253758

[ref66] Shu H.; Zhang R.; Xiao K.; Yang J.; Sun X. G-Quadruplex-Binding Proteins: Promising Targets for Drug Design. Biomolecules 2022, 12, 648 10.3390/biom12050648. 35625576 PMC9138358

[ref67] Wu Y.; Shin-ya K.; Brosh R. M. FANCJ Helicase Defective in Fanconia Anemia and Breast Cancer Unwinds G-Quadruplex DNA To Defend Genomic Stability. Mol. Cell. Biol. 2008, 28, 4116–4128. 10.1128/MCB.02210-07. 18426915 PMC2423121

[ref68] Postberg J.; Tsytlonok M.; Sparvoli D.; Rhodes D.; Lipps H. J. A telomerase-associated RecQ protein-like helicase resolves telomeric G-quadruplex structures during replication. Gene 2012, 497, 147–154. 10.1016/j.gene.2012.01.068. 22327026 PMC3650557

[ref69] Takahama K.; Takada A.; Tada S.; Shimizu M.; Sayama K.; Kurokawa R.; Oyoshi T. Regulation of Telomere Length by G-Quadruplex Telomere DNA- and TERRA-Binding Protein TLS/FUS. Chem. Biol. 2013, 20, 341–350. 10.1016/j.chembiol.2013.02.013. 23521792

[ref70] Iachettini S.; Ciccarone F.; Maresca C.; D’ Angelo C.; Petti E.; Di Vito S.; Ciriolo M. R.; Zizza P.; Biroccio A. The telomeric protein TERF2/TRF2 impairs HMGB1-driven autophagy. Autophagy 2023, 19, 1479–1490. 10.1080/15548627.2022.2138687. 36310382 PMC10240986

[ref71] Amato J.; Cerofolini L.; Brancaccio D.; Giuntini S.; Iaccarino N.; Zizza P.; Iachettini S.; Biroccio A.; Novellino E.; Rosato A.; Fragai M.; Luchinat C.; Randazzo A.; Pagano B. Insights into telomeric G-quadruplex DNA recognition by HMGB1 protein. Nucleic Acids Res. 2019, 47, 9950–9966. 10.1093/nar/gkz727. 31504744 PMC6765150

[ref72] Sánchez-Giraldo R.; Acosta-Reyes F. J.; Malarkey C. S.; Saperas N.; Churchill M. E. A.; Campos J. L. Two high-mobility group box domains act together to underwind and kink DNA. Acta Crystallogr. D Biol. Crystallogr. 2015, 71, 1423–1432. 10.1107/S1399004715007452. 26143914 PMC4498601

[ref73] Claussin C.; Chang M. The many facets of homologous recombination at telomeres. Microb. Cell 2015, 2, 308–321. 10.15698/mic2015.09.224. 28357308 PMC5354574

[ref74] Lee S.-H.; Princz L. N.; Klügel M. F.; Habermann B.; Pfander B.; Biertümpfel C. Human Holliday junction resolvase GEN1 uses a chromodomain for efficient DNA recognition and cleavage. eLife 2015, 4, e12256 10.7554/eLife.12256. 26682650 PMC5039027

[ref75] Sarkar J.; Wan B.; Yin J.; Vallabhaneni H.; Horvath K.; Kulikowicz T.; Bohr V. A.; Zhang Y.; Lei M.; Liu Y. SLX4 contributes to telomere preservation and regulated processing of telomeric joint molecule intermediates. Nucleic Acids Res. 2015, 43, 5912–5923. 10.1093/nar/gkv522. 25990736 PMC4499145

[ref76] Xu X.; Wang M.; Sun J.; Yu Z.; Li G.; Yang N.; Xu R.-M. Structure specific DNA recognition by the SLX1–SLX4 endonuclease complex. Nucleic Acids Res. 2021, 49, 7740–7752. 10.1093/nar/gkab542. 34181713 PMC8287910

[ref77] Sarek G.; Kotsantis P.; Ruis P.; Van Ly D.; Margalef P.; Borel V.; Zheng X.-F.; Flynn H. R.; Snijders A. P.; Chowdhury D.; Cesare A. J.; Boulton S. J. CDK phosphorylation of TRF2 controls t-loop dynamics during the cell cycle. Nature 2019, 575, 523–527. 10.1038/s41586-019-1744-8. 31723267 PMC6874499

[ref78] Uringa E.-J.; Youds J. L.; Lisaingo K.; Lansdorp P. M.; Boulton S. J. RTEL1: an essential helicase for telomere maintenance and the regulation of homologous recombination. Nucleic Acids Res. 2011, 39, 1647–1655. 10.1093/nar/gkq1045. 21097466 PMC3061057

[ref79] Kumar N.; Taneja A.; Ghosh M.; Rothweiler U.; Sundaresan N. R.; Singh M. Harmonin homology domain-mediated interaction of RTEL1 helicase with RPA and DNA provides insights into its recruitment to DNA repair sites. Nucleic Acids Res. 2024, 52, 1450–1470. 10.1093/nar/gkad1208. 38153196 PMC10853778

[ref80] Fasching C. L.; Cejka P.; Kowalczykowski S. C.; Heyer W.-D. Top3-Rmi1 Dissolve Rad51-Mediated D Loops by a Topoisomerase-Based Mechanism. Mol. Cell 2015, 57, 595–606. 10.1016/j.molcel.2015.01.022. 25699708 PMC4338411

[ref81] Piazza A.; Shah S. S.; Wright W. D.; Gore S. K.; Koszul R.; Heyer W.-D. Dynamic Processing of Displacement Loops during Recombinational DNA Repair. Mol. Cell 2019, 73, 1255–1266.e4. 10.1016/j.molcel.2019.01.005. 30737186 PMC6532985

[ref82] Pike A. C. W.; Gomathinayagam S.; Swuec P.; Berti M.; Zhang Y.; Schnecke C.; Marino F.; Von Delft F.; Renault L.; Costa A.; Gileadi O.; Vindigni A. Human RECQ1 helicase-driven DNA unwinding, annealing, and branch migration: Insights from DNA complex structures. Proc. Natl. Acad. Sci. U. S. A. 2015, 112, 4286–4291. 10.1073/pnas.1417594112. 25831490 PMC4394259

[ref83] Voter A. F.; Qiu Y.; Tippana R.; Myong S.; Keck J. L. A guanine-flipping and sequestration mechanism for G-quadruplex unwinding by RecQ helicases. Nat. Commun. 2018, 9, 4201 10.1038/s41467-018-06751-8. 30305632 PMC6180126

[ref84] Scully R.; Panday A.; Elango R.; Willis N. A. DNA double-strand break repair-pathway choice in somatic mammalian cells. Nat. Rev. Mol. Cell Biol. 2019, 20, 698–714. 10.1038/s41580-019-0152-0. 31263220 PMC7315405

[ref85] Menolfi D.; Zha S. ATM, ATR and DNA-PKcs kinases—the lessons from the mouse models: inhibition ≠ deletion. Cell Biosci. 2020, 10, 8 10.1186/s13578-020-0376-x. 32015826 PMC6990542

[ref86] Howes A. C.; Perisic O.; Williams R. L. Structural insights into the activation of ataxia-telangiectasia mutated by oxidative stress. Sci. Adv. 2023, 9, eadi8291 10.1126/sciadv.adi8291. 37756394 PMC10530080

[ref87] Rao Q.; Liu M.; Tian Y.; Wu Z.; Hao Y.; Song L.; Qin Z.; Ding C.; Wang H.-W.; Wang J.; Xu Y. Cryo-EM structure of human ATR-ATRIP complex. Cell Res. 2018, 28, 143–156. 10.1038/cr.2017.158. 29271416 PMC5799817

[ref88] Sharma S.; Anand R.; Zhang X.; Francia S.; Michelini F.; Galbiati A.; Williams H.; Ronato D. A.; Masson J.-Y.; Rothenberg E.; Cejka P.; d’Adda Di Fagagna F. MRE11-RAD50-NBS1 Complex Is Sufficient to Promote Transcription by RNA Polymerase II at Double-Strand Breaks by Melting DNA Ends. Cell Rep. 2021, 34, 108565 10.1016/j.celrep.2020.108565. 33406426 PMC7788559

[ref89] Helmink B. A.; Bredemeyer A. L.; Lee B.-S.; Huang C.-Y.; Sharma G. G.; Walker L. M.; Bednarski J. J.; Lee W.-L.; Pandita T. K.; Bassing C. H.; Sleckman B. P. MRN complex function in the repair of chromosomal Rag-mediated DNA double-strand breaks. J. Exp. Med. 2009, 206, 669–679. 10.1084/jem.20081326. 19221393 PMC2699138

[ref90] Williams G. J.; Williams R. S.; Williams J. S.; Moncalian G.; Arvai A. S.; Limbo O.; Guenther G.; SilDas S.; Hammel M.; Russell P.; Tainer J. A. ABC ATPase signature helices in Rad50 link nucleotide state to Mre11 interface for DNA repair. Nat. Struct. Mol. Biol. 2011, 18, 423–431. 10.1038/nsmb.2038. 21441914 PMC3118400

[ref91] Rotheneder M.; Stakyte K.; Van De Logt E.; Bartho J. D.; Lammens K.; Fan Y.; Alt A.; Kessler B.; Jung C.; Roos W. P.; Steigenberger B.; Hopfner K.-P. Cryo-EM structure of the Mre11-Rad50-Nbs1 complex reveals the molecular mechanism of scaffolding functions. Mol. Cell 2023, 83, 167–185.e9. 10.1016/j.molcel.2022.12.003. 36577401

[ref92] Bhattacharya S.; Srinivasan K.; Abdisalaam S.; Su F.; Raj P.; Dozmorov I.; Mishra R.; Wakeland E. K.; Ghose S.; Mukherjee S.; Asaithamby A. RAD51 interconnects between DNA replication, DNA repair and immunity. Nucleic Acids Res. 2017, 45, 4590–4605. 10.1093/nar/gkx126. 28334891 PMC5416901

[ref93] Feretzaki M.; Pospisilova M.; Valador Fernandes R.; Lunardi T.; Krejci L.; Lingner J. RAD51-dependent recruitment of TERRA lncRNA to telomeres through R-loops. Nature 2020, 587, 303–308. 10.1038/s41586-020-2815-6. 33057192 PMC7116795

[ref94] Xu J.; Zhao L.; Xu Y.; Zhao W.; Sung P.; Wang H.-W. Cryo-EM structures of human RAD51 recombinase filaments during catalysis of DNA-strand exchange. Nat. Struct. Mol. Biol. 2017, 24, 40–46. 10.1038/nsmb.3336. 27941862 PMC5471492

[ref95] Lockhart A.; Pires V. B.; Bento F.; Kellner V.; Luke-Glaser S.; Yakoub G.; Ulrich H. D.; Luke B. RNase H1 and H2 Are Differentially Regulated to Process RNA-DNA Hybrids. Cell Rep. 2019, 29, 2890–2900.e5. 10.1016/j.celrep.2019.10.108. 31775053

[ref96] Nowotny M.; Gaidamakov S. A.; Ghirlando R.; Cerritelli S. M.; Crouch R. J.; Yang W. Structure of Human RNase H1 Complexed with an RNA/DNA Hybrid: Insight into HIV Reverse Transcription. Mol. Cell 2007, 28, 264–276. 10.1016/j.molcel.2007.08.015. 17964265

[ref97] Reijns M. A. M.; Bubeck D.; Gibson L. C. D.; Graham S. C.; Baillie G. S.; Jones E. Y.; Jackson A. P. The Structure of the Human RNase H2 Complex Defines Key Interaction Interfaces Relevant to Enzyme Function and Human Disease. J. Biol. Chem. 2011, 286, 10530–10539. 10.1074/jbc.M110.177394. 21177854 PMC3060506

[ref98] Groh M.; Albulescu L. O.; Cristini A.; Gromak N. Senataxin: Genome Guardian at the Interface of Transcription and Neurodegeneration. J. Mol. Biol. 2017, 429, 3181–3195. 10.1016/j.jmb.2016.10.021. 27771483

[ref99] Cohen S.; Puget N.; Lin Y.-L.; Clouaire T.; Aguirrebengoa M.; Rocher V.; Pasero P.; Canitrot Y.; Legube G. Senataxin resolves RNA:DNA hybrids forming at DNA double-strand breaks to prevent translocations. Nat. Commun. 2018, 9, 533 10.1038/s41467-018-02894-w. 29416069 PMC5803260

[ref100] Chakraborty P.; Grosse F. Human DHX9 helicase preferentially unwinds RNA-containing displacement loops (R-loops) and G-quadruplexes. DNA Repair 2011, 10, 654–665. 10.1016/j.dnarep.2011.04.013. 21561811

[ref101] Lee Y.-T.; Sickmier E. A.; Grigoriu S.; Castro J.; Boriack-Sjodin P. A. Crystal structures of the DExH-box RNA helicase DHX9. Acta Crystallogr. Sect. Struct. Biol. 2023, 79, 980–991. 10.1107/S2059798323007611. PMC10619421 37860960

[ref102] Redon S.; Reichenbach P.; Lingner J. Protein–RNA and protein–protein interactions mediate association of human EST1A/SMG6 with telomerase. Nucleic Acids Res. 2007, 35, 7011–7022. 10.1093/nar/gkm724. 17940095 PMC2175296

[ref103] Isken O.; Maquat L. E. The multiple lives of NMD factors: balancing roles in gene and genome regulation. Nat. Rev. Genet. 2008, 9, 699–712. 10.1038/nrg2402. 18679436 PMC3711694

[ref104] Redon S.; Reichenbach P.; Lingner J. The non-coding RNA TERRA is a natural ligand and direct inhibitor of human telomerase. Nucleic Acids Res. 2010, 38, 5797–5806. 10.1093/nar/gkq296. 20460456 PMC2943627

[ref105] Glavan F.; Behm-Ansmant I.; Izaurralde E.; Conti E. Structures of the PIN domains of SMG6 and SMG5 reveal a nuclease within the mRNA surveillance complex. EMBO J. 2006, 25, 5117–5125. 10.1038/sj.emboj.7601377. 17053788 PMC1630413

[ref106] Yadav T.; Zhang J.-M.; Ouyang J.; Leung W.; Simoneau A.; Zou L. TERRA and RAD51AP1 promote alternative lengthening of telomeres through an R- to D-loop switch. Mol. Cell 2022, 82, 3985–4000.e4. 10.1016/j.molcel.2022.09.026. 36265486 PMC9637728

[ref107] Uziel O.; Yerushalmi R.; Zuriano L.; Naser S.; Beery E.; Nordenberg J.; Lubin I.; Adel Y.; Shepshelovich D.; Yavin H.; Aharon I. B.; Pery S.; Rizel S.; Pasmanik-Chor M.; Frumkin D.; Lahav M. BRCA1/2 mutations perturb telomere biology: characterization of structural and functional abnormalities in vitro and in vivo. Oncotarget 2016, 7, 2433–2454. 10.18632/oncotarget.5693. 26515461 PMC4823046

[ref108] Vohhodina J.; Goehring L. J.; Liu B.; Kong Q.; Botchkarev V. V.; Huynh M.; Liu Z.; Abderazzaq F. O.; Clark A. P.; Ficarro S. B.; Marto J. A.; Hatchi E.; Livingston D. M. BRCA1 binds TERRA RNA and suppresses R-Loop-based telomeric DNA damage. Nat. Commun. 2021, 12, 3542 10.1038/s41467-021-23716-6. 34112789 PMC8192922

[ref109] Zaug A. J.; Podell E. R.; Cech T. R. Human POT1 disrupts telomeric G-quadruplexes allowing telomerase extension in vitro. Proc. Natl. Acad. Sci. U. S. A. 2005, 102, 10864–10869. 10.1073/pnas.0504744102. 16043710 PMC1180509

[ref110] Traczyk A.; Liew C. W.; Gill D. J.; Rhodes D. Structural basis of G-quadruplex DNA recognition by the yeast telomeric protein Rap1. Nucleic Acids Res. 2020, 48, 4562–4571. 10.1093/nar/gkaa171. 32187364 PMC7192608

[ref111] De Boeck G.; Forsyth R. G.; Praet M.; Hogendoorn P. C. Telomere-associated proteins: cross-talk between telomere maintenance and telomere-lengthening mechanisms: Cross-talk between telomere maintenance and lengthening mechanisms. J. Pathol. 2009, 217, 327–344. 10.1002/path.2500. 19142887

[ref112] Haider S.; Parkinson G. N.; Neidle S. Crystal Structure of the Potassium Form of an Oxytricha nova G-quadruplex. J. Mol. Biol. 2002, 320, 189–200. 10.1016/S0022-2836(02)00428-X. 12079378

[ref113] Parkinson G. N.; Lee M. P. H.; Neidle S. Crystal structure of parallel quadruplexes from human telomeric DNA. Nature 2002, 417, 876–880. 10.1038/nature755. 12050675

[ref114] Neidle S.; Parkinson G. N. Quadruplex DNA crystal structures and drug design. Biochimie 2008, 90, 1184–1196. 10.1016/j.biochi.2008.03.003. 18395014

[ref115] Frasson I.; Pirota V.; Richter S. N.; Doria F. Multimeric G-quadruplexes: A review on their biological roles and targeting. Int. J. Biol. Macromol. 2022, 204, 89–102. 10.1016/j.ijbiomac.2022.01.197. 35124022

[ref116] Haider S.; Parkinson G. N.; Neidle S. Molecular Dynamics and Principal Components Analysis of Human Telomeric Quadruplex Multimers. Biophys. J. 2008, 95, 296–311. 10.1529/biophysj.107.120501. 18375510 PMC2426654

[ref117] Monsen R. C.; Trent J. O.; Chaires J. B. G-quadruplex DNA: A Longer Story. Acc. Chem. Res. 2022, 55, 3242–3252. 10.1021/acs.accounts.2c00519. 36282946

[ref118] Ahmed A. A.; Chen S.; Roman-Escorza M.; Angell R.; Oxenford S.; McConville M.; Barton N.; Sunose M.; Neidle D.; Haider S.; Arshad T.; Neidle S. Structure–activity relationships for the G-quadruplex-targeting experimental drug QN-302 and two analogues probed with comparative transcriptome profiling and molecular modeling. Sci. Rep. 2024, 14, 3447 10.1038/s41598-024-54080-2. 38342953 PMC10859377

[ref119] Marchetti C.; Zyner K. G.; Ohnmacht S. A.; Robson M.; Haider S. M.; Morton J. P.; Marsico G.; Vo T.; Laughlin-Toth S.; Ahmed A. A.; Di Vita G.; Pazitna I.; Gunaratnam M.; Besser R. J.; Andrade A. C. G.; Diocou S.; Pike J. A.; Tannahill D.; Pedley R. B.; Evans T. R. J.; Wilson W. D.; Balasubramanian S.; Neidle S. Targeting Multiple Effector Pathways in Pancreatic Ductal Adenocarcinoma with a G-Quadruplex-Binding Small Molecule. J. Med. Chem. 2018, 61, 2500–2517. 10.1021/acs.jmedchem.7b01781. 29356532 PMC5867665

[ref120] Sanchez-Martin V. DNA G-Quadruplex-Binding Proteins: An Updated Overview. DNA 2023, 3, 1–12. 10.3390/dna3010001.

[ref121] Wu C. G.; Spies M. G-quadruplex recognition and remodeling by the FANCJ helicase. Nucleic Acids Res. 2016, 44, 8742–8753. 10.1093/nar/gkw574. 27342280 PMC5062972

[ref122] Lowran K.; Campbell L.; Popp P.; Wu C. G. Assembly of a G-Quadruplex Repair Complex by the FANCJ DNA Helicase and the REV1 Polymerase. Genes 2020, 11, 5 10.3390/genes11010005. PMC7017153 31861576

[ref123] Lemmens B.; Van Schendel R.; Tijsterman M. Mutagenic consequences of a single G-quadruplex demonstrate mitotic inheritance of DNA replication fork barriers. Nat. Commun. 2015, 6, 8909 10.1038/ncomms9909. 26563448 PMC4654259

[ref124] Osmundson J. S.; Kumar J.; Yeung R.; Smith D. J. Pif1-family helicases cooperatively suppress widespread replication-fork arrest at tRNA genes. Nat. Struct. Mol. Biol. 2017, 24, 162–170. 10.1038/nsmb.3342. 27991904 PMC5296403

[ref125] Wei C.; Price M. Protecting the terminus: t-loops and telomere end-binding proteins. Cell. Mol. Life Sci. CMLS 2003, 60, 2283–2294. 10.1007/s00018-003-3244-z. 14625675 PMC11138721

[ref126] Tomaska L.; Nosek J.; Kar A.; Willcox S.; Griffith J. D. A New View of the T-Loop Junction: Implications for Self-Primed Telomere Extension, Expansion of Disease-Related Nucleotide Repeat Blocks, and Telomere Evolution. Front. Genet. 2019, 10, 792 10.3389/fgene.2019.00792. 31475042 PMC6702307

[ref127] Spies M.; Fishel R. Mismatch Repair during Homologous and Homeologous Recombination. Cold Spring Harb. Perspect. Biol. 2015, 7, a022657 10.1101/cshperspect.a022657. 25731766 PMC4355274

[ref128] Kong C. M.; Lee X. W.; Wang X. Telomere shortening in human diseases. FEBS J. 2013, 280, 3180–3193. 10.1111/febs.12326. 23647631

[ref129] Kar A.; Willcox S.; Griffith J. D. Transcription of telomeric DNA leads to high levels of homologous recombination and t-loops. Nucleic Acids Res. 2016, gkw779 10.1093/nar/gkw779. PMC5100571 27608724

[ref130] Mazzucco G.; Huda A.; Galli M.; Piccini D.; Giannattasio M.; Pessina F.; Doksani Y. Telomere damage induces internal loops that generate telomeric circles. Nat. Commun. 2020, 11, 5297 10.1038/s41467-020-19139-4. 33082350 PMC7576219

[ref131] Recagni M.; Bidzinska J.; Zaffaroni N.; Folini M. The Role of Alternative Lengthening of Telomeres Mechanism in Cancer: Translational and Therapeutic Implications. Cancers 2020, 12, 949 10.3390/cancers12040949. 32290440 PMC7226354

[ref132] Santos-Pereira J. M.; Aguilera A. R loops: new modulators of genome dynamics and function. Nat. Rev. Genet. 2015, 16, 583–597. 10.1038/nrg3961. 26370899

[ref133] Allison D. F.; Wang G. G. R-loops: formation, function, and relevance to cell stress. Cell Stress 2019, 3, 38–46. 10.15698/cst2019.02.175. 31225499 PMC6551709

[ref134] Freudenreich C. H. R-loops: targets for nuclease cleavage and repeat instability. Curr. Genet. 2018, 64, 789–794. 10.1007/s00294-018-0806-z. 29327083 PMC6039234

[ref135] Sollier J.; Cimprich K. A. Breaking bad: R-loops and genome integrity. Trends Cell Biol. 2015, 25, 514–522. 10.1016/j.tcb.2015.05.003. 26045257 PMC4554970

[ref136] Yang S. Y.; Chang E. Y. C.; Lim J.; Kwan H. H.; Monchaud D.; Yip S.; Stirling P. C.; Wong J. M. Y. G-quadruplexes mark alternative lengthening of telomeres. NAR Cancer 2021, 3, zcab031 10.1093/narcan/zcab031. 34316718 PMC8294677

[ref137] Duquette M. L.; Handa P.; Vincent J. A.; Taylor A. F.; Maizels N. Intracellular transcription of G-rich DNAs induces formation of G-loops, novel structures containing G4 DNA. Genes Dev. 2004, 18, 1618–1629. 10.1101/gad.1200804. 15231739 PMC443523

[ref138] Bettin N.; Oss Pegorar C.; Cusanelli E. The Emerging Roles of TERRA in Telomere Maintenance and Genome Stability. Cells 2019, 8, 246 10.3390/cells8030246. 30875900 PMC6468625

[ref139] Rivosecchi J.; Jurikova K.; Cusanelli E. Telomere-specific regulation of TERRA and its impact on telomere stability. Semin. Cell Dev. Biol. 2024, 157, 3–23. 10.1016/j.semcdb.2023.11.001. 38088000

[ref140] Wang C.; Zhao L.; Lu S. Role of TERRA in the Regulation of Telomere Length. Int. J. Biol. Sci. 2015, 11, 316–323. 10.7150/ijbs.10528. 25678850 PMC4323371

[ref141] Chawla R.; Azzalin C. M. The telomeric transcriptome and SMG proteins at the crossroads. Cytogenet. Genome Res. 2009, 122, 194–201. 10.1159/000167804. 19188687

[ref142] Fiset S. hnRNP A1 may interact simultaneously with telomeric DNA and the human telomerase RNA in vitro. Nucleic Acids Res. 2001, 29, 2268–2275. 10.1093/nar/29.11.2268. 11376145 PMC55710

[ref143] Hatchi E.; Goehring L.; Landini S.; Skourti-Stathaki K.; DeConti D. K.; Abderazzaq F. O.; Banerjee P.; Demers T. M.; Wang Y. E.; Quackenbush J.; Livingston D. M. BRCA1 and RNAi factors promote repair mediated by small RNAs and PALB2–RAD52. Nature 2021, 591, 665–670. 10.1038/s41586-020-03150-2. 33536619 PMC8245199

[ref144] Ouyang J.; Yadav T.; Zhang J.-M.; Yang H.; Rheinbay E.; Guo H.; Haber D. A.; Lan L.; Zou L. RNA transcripts stimulate homologous recombination by forming DR-loops. Nature 2021, 594, 283–288. 10.1038/s41586-021-03538-8. 33981036 PMC8855348

[ref145] Guéron M.; Leroy J.-L. The i-motif in nucleic acids. Curr. Opin. Struct. Biol. 2000, 10, 326–331. 10.1016/S0959-440X(00)00091-9. 10851195

[ref146] Guneri D.; Alexandrou E.; El Omari K.; Dvořáková Z.; Chikhale R. V.; Pike D.; Waudby C. A.; Morris C. J.; Haider S.; Parkinson G. N.; Waller Z. A. E. Structural Insights into Regulation of Insulin Expression Involving i-Motif DNA Structures in the Insulin-Linked Polymorphic Region. BioRxiv. 2023. 10.1038/s41467-024-50553-0 PMC11336075 39164244

[ref147] Zeraati M.; Langley D. B.; Schofield P.; Moye A. L.; Rouet R.; Hughes W. E.; Bryan T. M.; Dinger M. E.; Christ D. I-motif DNA structures are formed in the nuclei of human cells. Nat. Chem. 2018, 10, 631–637. 10.1038/s41557-018-0046-3. 29686376

[ref148] Abou Assi H.; Garavís M.; González C.; Damha M. J. i-Motif DNA: structural features and significance to cell biology. Nucleic Acids Res. 2018, 46, 8038–8056. 10.1093/nar/gky735. 30124962 PMC6144788

[ref149] Malliavin T. E.; Gau J.; Snoussi K.; Leroy J.-L. Stability of the I-motif Structure Is Related to the Interactions between Phosphodiester Backbones. Biophys. J. 2003, 84, 3838–3847. 10.1016/S0006-3495(03)75111-X. 12770889 PMC1302965

[ref150] Wright E. P.; Huppert J. L.; Waller Z. A. E. Identification of multiple genomic DNA sequences which form i-motif structures at neutral pH. Nucleic Acids Res. 2017, 45, 2951–2959. 10.1093/nar/gkx090. 28180276 PMC5605235

[ref151] Wright E. P.; Abdelhamid M. A. S.; Ehiabor M. O.; Grigg M. C.; Irving K.; Smith N. M.; Waller Z. A. E. Epigenetic modification of cytosines fine tunes the stability of i-motif DNA. Nucleic Acids Res. 2020, 48, 55–62. 10.1093/nar/gkz1082. 31777919 PMC6943138

[ref152] Gurung S. P.; Schwarz C.; Hall J. P.; Cardin C. J.; Brazier J. A. The importance of loop length on the stability of i-motif structures. Chem. Commun. 2015, 51, 5630–5632. 10.1039/C4CC07279K. PMC4384421 25686374

[ref153] Bhavsar-Jog Y. P.; Van Dornshuld E.; Brooks T. A.; Tschumper G. S.; Wadkins R. M. Epigenetic Modification, Dehydration, and Molecular Crowding Effects on the Thermodynamics of i-Motif Structure Formation from C-Rich DNA. Biochemistry 2014, 53, 1586–1594. 10.1021/bi401523b. 24564458 PMC3985701

[ref154] Xu B.; Devi G.; Shao F. Regulation of telomeric i-motif stability by 5-methylcytosine and 5-hydroxymethylcytosine modification. Org. Biomol. Chem. 2015, 13, 5646–5651. 10.1039/C4OB02646B. 25886653

[ref155] Amato J.; D’Aria F.; Marzano S.; Iaccarino N.; Randazzo A.; Giancola C.; Pagano B. On the thermodynamics of folding of an i-motif DNA in solution under favorable conditions. Phys. Chem. Chem. Phys. 2021, 23, 15030–15037. 10.1039/D1CP01779A. 34151914

[ref156] Tsvetkov V. B.; Zatsepin T. S.; Belyaev E. S.; Kostyukevich Y. I.; Shpakovski G. V.; Podgorsky V. V.; Pozmogova G. E.; Varizhuk A. M.; Aralov A. V. i-Clamp phenoxazine for the fine tuning of DNA i-motif stability. Nucleic Acids Res. 2018, 46, 2751–2764. 10.1093/nar/gky121. 29474573 PMC5888743

[ref157] Školáková P.; Renčiuk D.; Palacký J.; Krafčík D.; Dvořáková Z.; Kejnovská I.; Bednářová K.; Vorlíčková M. Systematic investigation of sequence requirements for DNA i-motif formation. Nucleic Acids Res. 2019, 47, 2177–2189. 10.1093/nar/gkz046. 30715498 PMC6412112

[ref158] Assi H. A.; Harkness R. W.; Martin-Pintado N.; Wilds C. J.; Campos-Olivas R.; Mittermaier A. K.; González C.; Damha M. J. Stabilization of i-motif structures by 2′-β-fluorination of DNA. Nucleic Acids Res. 2016, 44, 4998–5009. 10.1093/nar/gkw402. 27166371 PMC4914123

[ref159] Abdelhamid M. A. S.; Waller Z. A. E. Tricky Topology: Persistence of Folded Human Telomeric i-Motif DNA at Ambient Temperature and Neutral pH. Front. Chem. 2020, 8, 40 10.3389/fchem.2020.00040. 32083057 PMC7005205

[ref160] Sun D.; Hurley L. H. The Importance of Negative Superhelicity in Inducing the Formation of G-Quadruplex and i-Motif Structures in the c-Myc Promoter: Implications for Drug Targeting and Control of Gene Expression. J. Med. Chem. 2009, 52, 2863–2874. 10.1021/jm900055s. 19385599 PMC2757002

[ref161] Irving K. L.; King J. J.; Waller Z. A. E.; Evans C. W.; Smith N. M. Stability and context of intercalated motifs (i-motifs) for biological applications. Biochimie 2022, 198, 33–47. 10.1016/j.biochi.2022.03.001. 35259471

[ref162] Lilley D. M. J. Structures of helical junctions in nucleic acids. Q. Rev. Biophys. 2000, 33, 109–159. 10.1017/S0033583500003590. 11131562

[ref163] Polleys E. J.; Freudenreich C. H. Homologous recombination within repetitive DNA. Curr. Opin. Genet. Dev. 2021, 71, 143–153. 10.1016/j.gde.2021.08.005. 34464817 PMC8671197

[ref164] Symington L. S.; Rothstein R.; Lisby M. Mechanisms and Regulation of Mitotic Recombination in Saccharomyces cerevisiae. Genetics 2014, 198, 795–835. 10.1534/genetics.114.166140. 25381364 PMC4224172

[ref165] Li X.; Heyer W.-D. Homologous recombination in DNA repair and DNA damage tolerance. Cell Res. 2008, 18, 99–113. 10.1038/cr.2008.1. 18166982 PMC3087377

[ref166] Haider S.; Li P.; Khiali S.; Munnur D.; Ramanathan A.; Parkinson G. N. Holliday Junctions Formed from Human Telomeric DNA. J. Am. Chem. Soc. 2018, 140, 15366–15374. 10.1021/jacs.8b08699. 30376323

[ref167] Duckett D. R.; Murchie A. I. H.; Diekmann S.; Von Kitzing E.; Kemper B.; Lilley D. M. J. The structure of the holliday junction, and its resolution. Cell 1988, 55, 79–89. 10.1016/0092-8674(88)90011-6. 3167979

[ref168] Duckett D. R.; Murchie A. I.; Lilley D. M. The role of metal ions in the conformation of the four-way DNA junction. EMBO J. 1990, 9, 583–590. 10.1002/j.1460-2075.1990.tb08146.x. 2303044 PMC551705

[ref169] Bryan T. M.; Cech T. R. Telomerase and the maintenance of chromosome ends. Curr. Opin. Cell Biol. 1999, 11, 318–324. 10.1016/S0955-0674(99)80043-X. 10395557

[ref170] Shah Punatar R.; Martin M. J.; Wyatt H. D. M.; Chan Y. W.; West S. C. Resolution of single and double Holliday junction recombination intermediates by GEN1. Proc. Natl. Acad. Sci. U. S. A. 2017, 114, 443–450. 10.1073/pnas.1619790114. 28049850 PMC5255610

[ref171] Watson J. Definitions and analysis of DNA Holliday junction geometry. Nucleic Acids Res. 2004, 32, 3017–3027. 10.1093/nar/gkh631. 15173384 PMC434437

[ref172] Bizard A. H.; Hickson I. D. The Dissolution of Double Holliday Junctions. Cold Spring Harb. Perspect. Biol. 2014, 6, a016477–a016477. 10.1101/cshperspect.a016477. 24984776 PMC4067992

[ref173] Wyatt H. D. M.; West S. C. Holliday Junction Resolvases. Cold Spring Harb. Perspect. Biol. 2014, 6, a023192–a023192. 10.1101/cshperspect.a023192. 25183833 PMC4142969

[ref174] Hoang S. M.; O’Sullivan R. J. Alternative Lengthening of Telomeres: Building Bridges To Connect Chromosome Ends. Trends Cancer 2020, 6, 247–260. 10.1016/j.trecan.2019.12.009. 32101727 PMC7199893

